# Bibliometric analysis of kinship analysis from 1960 to 2023: global trends and development

**DOI:** 10.3389/fgene.2024.1401898

**Published:** 2024-06-06

**Authors:** Yanchen Liu, Chaoran Sun, Haoyuan Si, Zixuan Peng, Liya Gu, Xiangnan Guo, Feng Song

**Affiliations:** Department of Forensic Genetics, West China School of Basic Medical Sciences & Forensic Medicine, Sichuan University, Chengdu, China

**Keywords:** bibliometric analysis, kinship analysis, forensic genetics, research trend, forensic genealogy

## Abstract

Kinship analysis is a crucial aspect of forensic genetics. This study analyzed 1,222 publications on kinship analysis from 1960 to 2023 using bibliometric analysis techniques, investigating the annual publication and citation patterns, most productive countries, organizations, authors and journals, most cited documents and co-occurrence of keywords. The initial publication in this field occurred in 1960. Since 2007, there has been a significant increase in publications, with over 30 published annually except for 2010. China had the most publications (*n* = 213, 17.43%), followed by the United States (*n* = 175, 14.32%) and Germany (*n* = 89, 7.28%). The United States also had the highest citation count. Sichuan University in China has the largest number of published articles. The University of Leipzig and the University of Cologne in Germany exhibit the highest total citation count and average citation, respectively. Budowle B was the most prolific author and Kayser M was the most cited author. In terms of publications, *Forensic Science* *International-* *Genetics*, *Forensic Science International*, and *International Journal of Legal Medicine* were the most prolific journals. Among them, *Forensic Science International-Genetics* boasted the highest h-index, citation count, and average citation rate. The most frequently cited publication was “Van Oven M, 2009, Hum Mutat”, with a total of 1,361 citations. The most frequent co-occurrence keyword included “DNA”, “Loci”, “Paternity testing”, “Population”, “Markers”, and “Identification”, with recent interest focusing on “Kinship analysis”, “SNP” and “Inference”. The current research is centered around microhaplotypes, forensic genetic genealogy, and massively parallel sequencing. The field advanced with new DNA analysis methods, tools, and genetic markers. Collaborative research among nations, organizations, and authors benefits idea exchange, problem-solving efficiency, and high-quality results.

## 1 Introduction

Kinship analysis involves determining the presence of a certain kinship relationship between individuals by examining the genetic markers through testing, based on the principles of heredity (Weir et al., 2006). Kinship analysis in the past focused mostly on paternity tests to confirm the father-child link.

**FIGURE 1:**
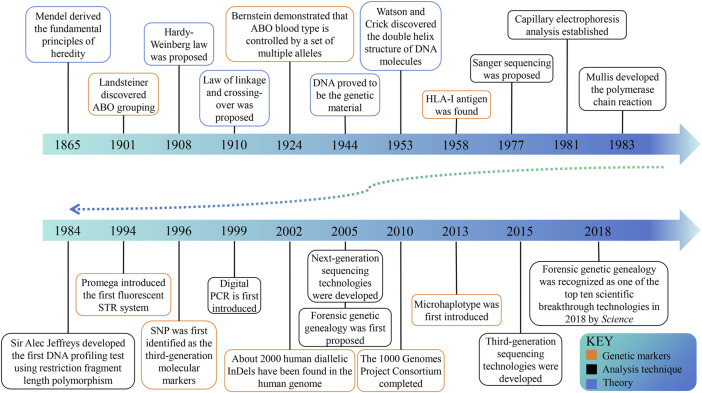
The historical timeline on milestones in kinship analysis. The progress of genetic theory lays the foundation for kinship analysis. With the discovery of genetic markers and the progress of analysis technology, the field is developing continuously.

In ancient times, various methods were used to identify kinship, but there was no scientific evidence to confirm their accuracy (Silver, 1989). The first scientific approach to paternity testing can be attributed to the identification of blood grouping (Figure 1) (Landsteiner, 1900; von Dungern and Hirschfeld, 1962), and in 1926 Austria pioneered the acceptance of forensic serology as admissible evidence in paternity testing cases (Mayr et al., 1991). The genetic indicators of the second-generation paternity testing are serum protein and erythrocyte enzyme isoenzymes to address the difficulties caused by a significant number of blood group similarities (Smithies, 1955; Hirschfeld et al., 1960; Dykes and Polesky, 1976). The discovery of highly efficient HLA-I antigen signifies the emergence of the third generation identification technology in 1958 (Dausset, 1958). In the early 1980s, the fourth generation emerged (Walker and Crisan, 1991), utilizing DNA probes to detect restriction fragment length polymorphisms (RFLPs) (Southern, 1975). In the subsequent decade, the development of polymerase chain reaction (PCR) (Saiki et al., 1988) and capillary electrophoresis (CE) has propelled short tandem repeat (STR) length polymorphism to the forefront of forensic genetics. The utilization of STR’s capillary electrophoresis technique has proven to be precise, cost-effective (Baine and Hui, 2019), and streamlined, rapidly establishing itself as the predominant method worldwide. With the advancement of DNA sequencing technologies, such as microarray genotyping (Amorim and Pereira, 2005) and next-generation sequencing (NGS) (Reis-Filho, 2009), the exploration of DNA sequence polymorphisms including single nucleotide polymorphisms (SNPs) and microhaplotypes (MHs) has progressively increased. These methodologies complement STR analysis (Butler, 2007; Tam et al., 2020), but their widespread adoption has been hindered by high costs. Currently, CE-based STR analysis remains the gold standard for paternity testing in forensic DNA analysis (Butler et al., 2004).

Forensic kinship research has flourished in recent years, mainly based on the expansion of genetic markers and sample size in relevant studies (Bertoglio et al., 2020; Alterauge et al., 2021; Zou et al., 2022; Cui et al., 2023). Although CE is a convenient and cost-effective approach for detecting STR loci, STR loci high mutation rate, long amplified fragments, and limited number of loci restrict their use in complex kinship analysis. (Alterauge et al., 2021; Zhang et al., 2022; Cui et al., 2023). SNPs and insertions-deletions (InDels) have shown more obvious advantages in complex kinship analysis (Nothnagel et al., 2010; Wu et al., 2021; Zhang et al., 2022). Zhu et al. used the MGISEQ-2000RS platform to sequence 1993 SNP loci in 119 Chinese Han individuals from eight families and found that the panel could be applied in paternity testing, full sibling testing, second-degree kinship, and first cousin kinship analyses (Cui et al., 2023). Liang et al. performed a genome-wide screen for new MH markers consisting of two or more variants (InDels or SNPs) within 220 bp and successfully developed an NGS-based 67plex MH panel to complement complex kinship analysis (Xue et al., 2023). The availability of human genetic data has significantly increased due to the commercialization of DNA testing and the public’s interest in their DNA and genetic ancestry (Phillips, 2018; Glynn, 2022; Snedecor et al., 2022; Tvedebrink, 2022). This has led to the emergence of forensic genetic genealogy (FGG). In 2018, the investigation of the Golden State Killer case in the United States opened the door to the application of FGG technology and was named one of the top ten scientific breakthroughs of the year by *Science*, which garnered significant attention within the domain of kinship analysis (Kaiser, 2018; Phillips, 2018; Ram and Roberts, 2019; Glynn, 2022).

Kinship analysis is one of the main tasks of forensic science. The scope of kinship analysis has expanded from the conventional parent-child relationship (usually father-child relationship) to the complex kinship analysis such as full sibling, great-grandson and half-sibling (Cui et al., 2023). kinship analysis plays an important role in inheritance disputes, disaster victim identification, and criminal investigations (Bertoglio et al., 2020; Alterauge et al., 2021). The identification of blood types has evaluated the kinship analysis into the realm of science. With the emergence of PCR and CE techniques, the use of STR profiles based on DNA length polymorphisms can accurately determine genetic relationships between individuals. NGS technology has enabled the extensive use of new genetic markers such as SNP and MH, which are based on DNA sequence polymorphisms, in complex kinship analysis. In recent years, research in this field has boomed, and this paper aims to describe the general situation of kinship analysis through bibliometric analysis. This study used the Web of Science Core Collection database to conduct a bibliometrics analysis of relevant literature in the field of kinship analysis from 1960 to 2023, with the aim of identifying the most affected countries, authors, and evaluating current research directions in this field.

## 2 Methods

### 2.1 Database and search strategy

We performed a literature search using the Web of Science (WoS) Core Collection (Science Citation Index Expanded) Database on 23 January 2024, covering literature published from 1960 to 2023.

**FIGURE 2:**
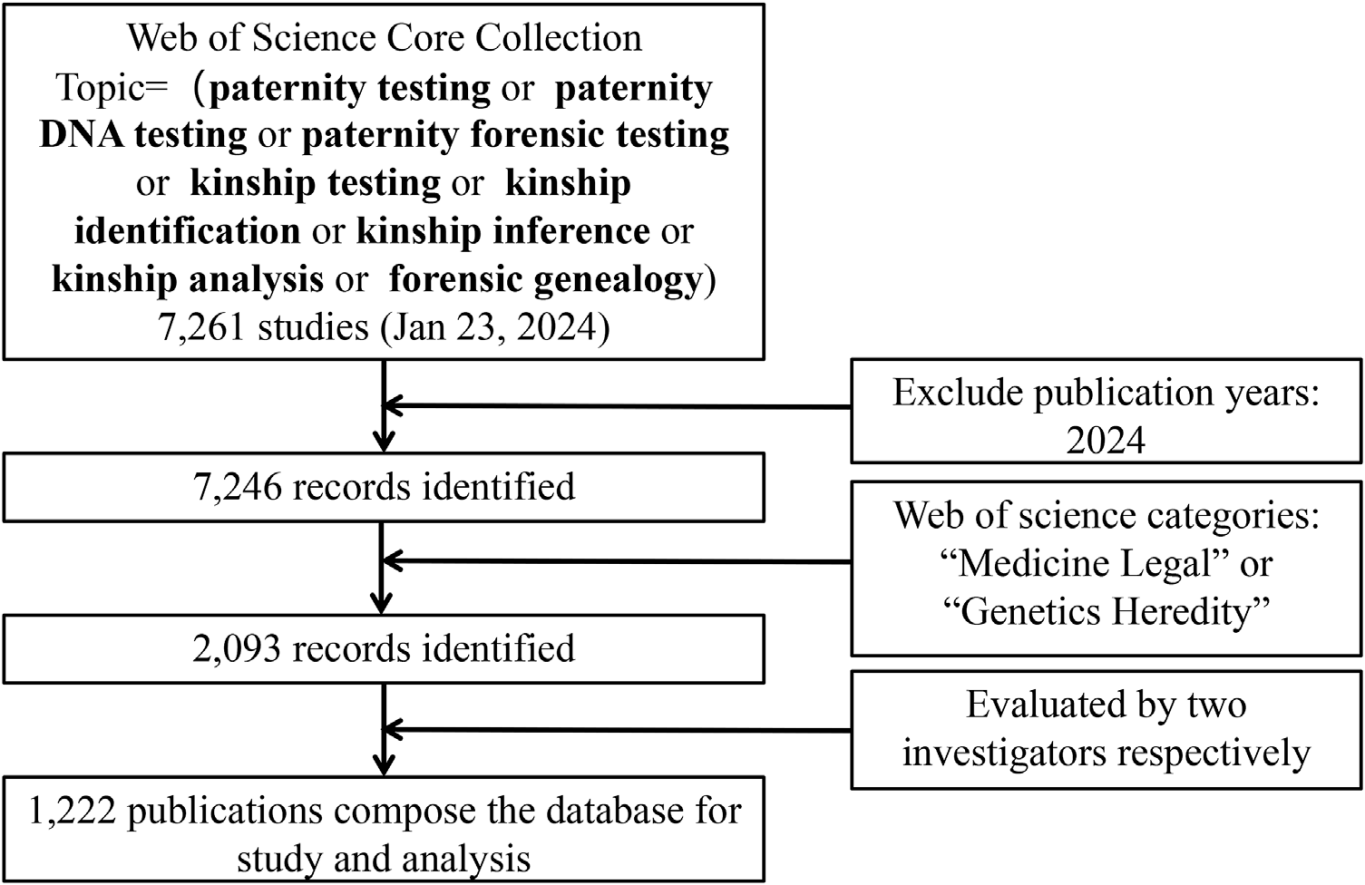
Search strategy.

The search strategy is as follows. First, the term and topic paternity testing, paternity DNA testing, paternity forensic testing, kinship testing, kinship identification, kinship inference, kinship analysis, forensic genealogy were searched in the WoS Core Collection. All categories of publications were considered, and no time restrictions were placed. A total of 7,261 papers were retrieved, of which 7,246 reports were dated before 2024. Secondly, we limited the publications in the field of kinship analysis to all those indexed under the research category “Medicine Legal” or “Genetics Heredity” in the WoS database and identified 2,093 papers. Two independent investigators evaluated all documents, focusing on titles and abstracts to verify that the documents were related to kinship analysis. If necessary, the investigators read the full text to decide on inclusion. Finally, 1,222 papers were included and exported from the WoS (Figure 2).

### 2.2 Data analysis and visualization

Bibliometric analysis of 1,222 documents were performed using VOSviewer (version 1.6.19) software, open-source Biblioshiny (RStudio) and MS Excel. VOSviewer is a bibliometric network builder and visualization software based on publication, country, author, journal and keywords (Van Eck and Waltman, 2010). Biblioshiny has a graphical interface and a complete bibliometric and visualization methodology, which is quite useful for bibliometric analysis.

To analyze the basic trend of the articles in kinship analysis, the following indicators were selected: the annual scientific productivity, top contributing countries and organizations, top 20 productive authors, top 20 journals contributed to publications, top 20 cited articles, top 20 co-occurrence keywords, and the change of topics. The Three-field plot analysis representing author, country and source relationship was compiled using Biblioshiny. In order to better evaluate the level of researchers, we introduced h-index, g-index and m-index. The h-index, defined as the maximum value of h, where an author has published at least h papers, each being cited at least h times, is a measure of academic impact. For example, if an author has published 10 papers, and each of them has been cited at least 10 times, their h-index would be 10. The g-index supplements the h-index by taking into account the citation counts of highly cited papers, which is a measure that calculates the top g articles having at least g^2^ citations. The m-index is defined as h/n, where h represents the h-index and n represents the number of years since the scientist’s first published paper. The m-index considers the impact of scholars’ varying ages on citation counts.

## 3 Result

### 3.1 The annual trends in growth and average citations of publications

**FIGURE 3:**
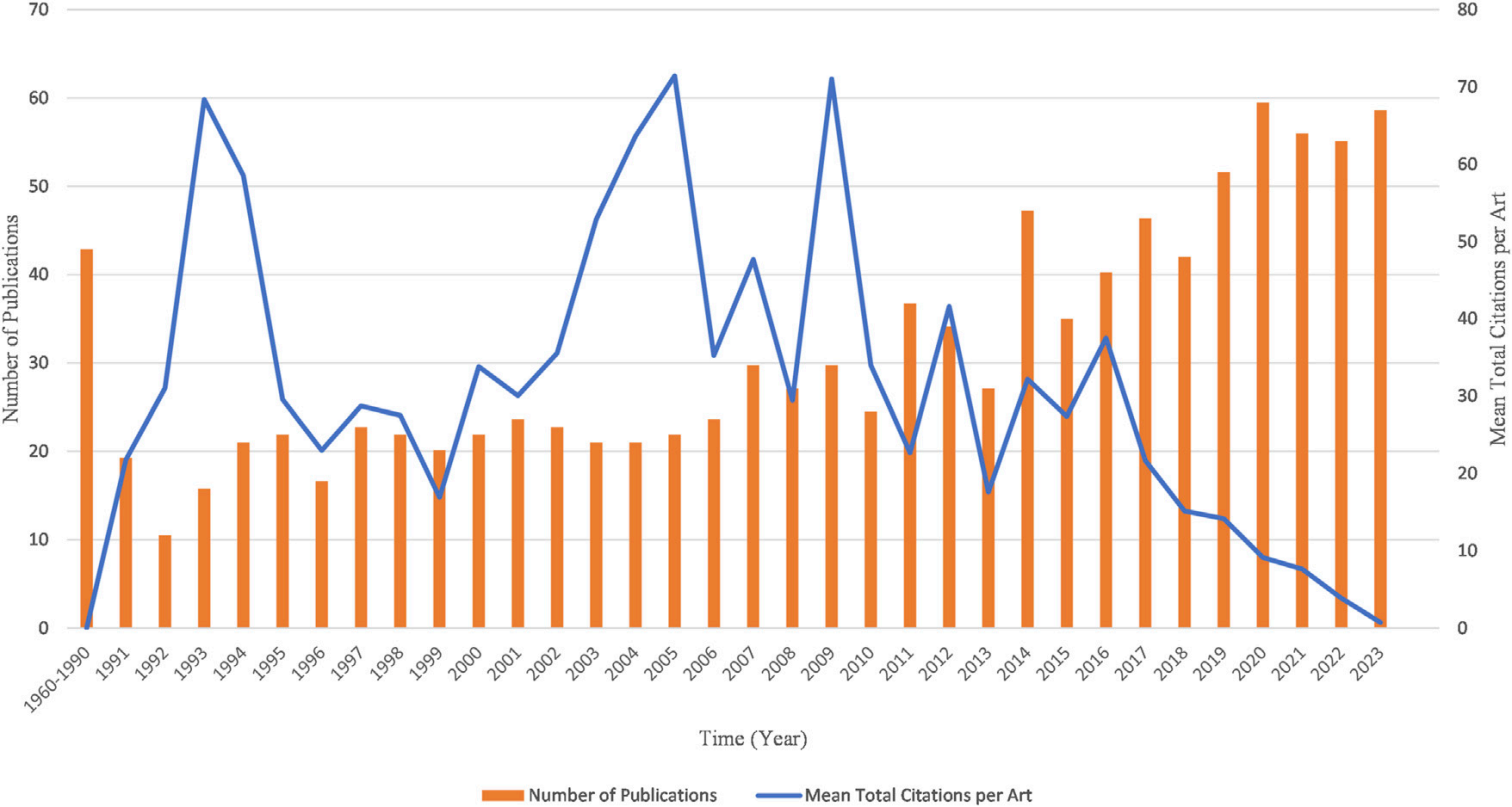
Chronological distribution of publications and mean total citation per publication in the field of kinship analysis.

Counting the number of publications and analyzing the development trend can help predict the future direction of kinship analysis. This field has attracted great interest among researchers worldwide. The first publication in this field was published in 1960, and except for one publication in 1969, no more publications were published until 1973 (Figure 3). From 1960 to 1990, there were no more than 10 publications published each year. However, since 2007, there has been a considerable increase in the number of publications, with more than 30 publications published each year except for 2010. The year 2020 had the highest number of publications with 68 publications, followed by 64, 64, and 63 publications in 2023, 2021 and 2022. The highest mean citations were in 2005 (62.52 citations per publication), followed by 2009, 1993, with 62.15 and 59.83 citations per year respectively (Figure 3). In other periods, the mean citations remained lower than 50.

### 3.2 Countries and organizations that make the greatest contribution

**FIGURE 4:**
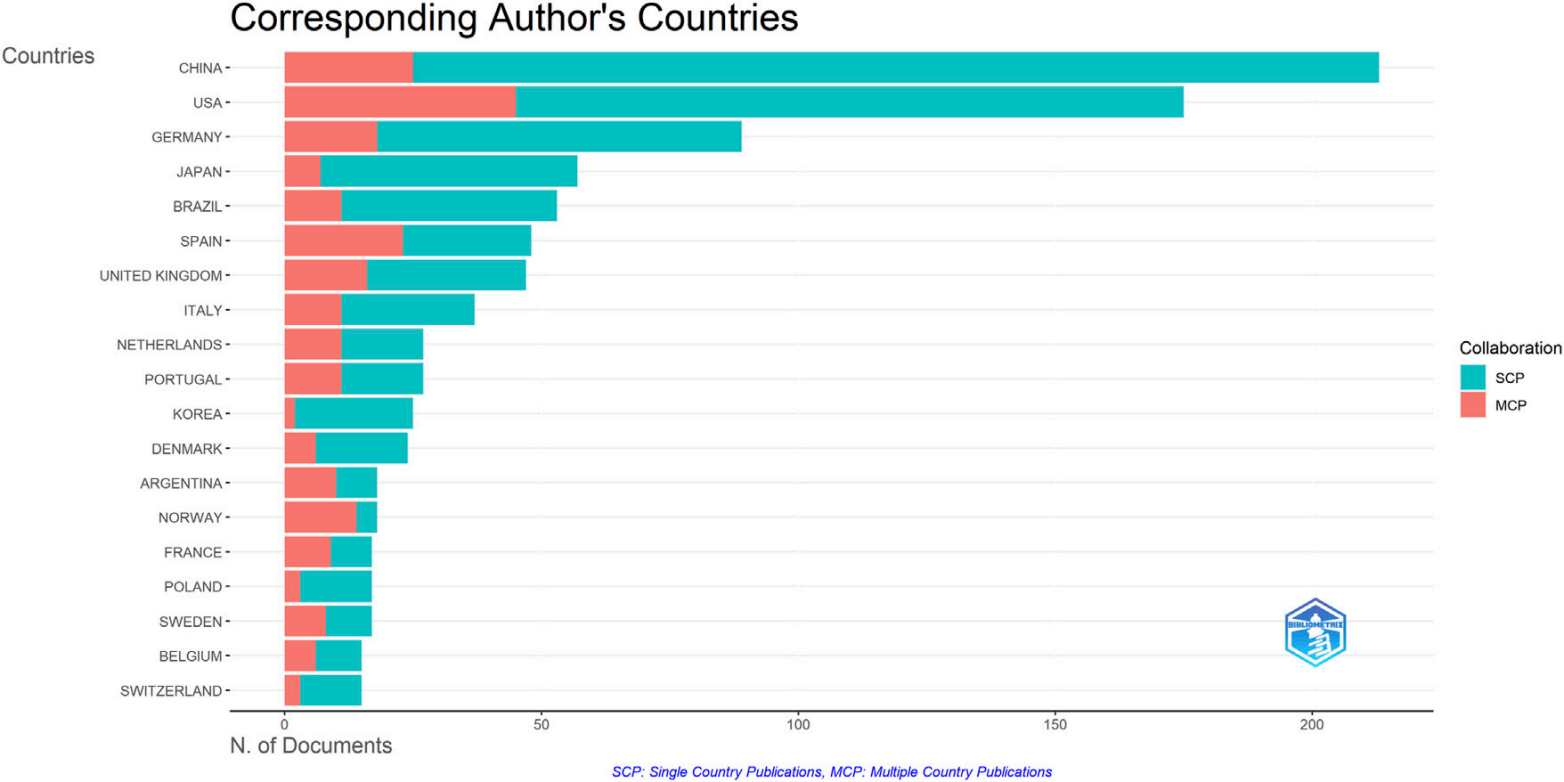
Inter and Intra collaboration of various countries. MCP indicates collaboration among different countries, while SCP indicates the production of a single country. Countries were selected based on the corresponding author’s country.

A total of 1,222 publications were published in the field of kinship analysis, with China having the highest number of publications at 213, accounting for 17.43% (Table 1). The United States was next with 175 (14.32**%**) publications, followed by Germany (89, accounting for 7.28%), Japan (57, accounting for 4.66%), Brazil (53, accounting for 4.34%), Spain (48, accounting for 3.93%), The United Kingdom (47, accounting for 3.85%), Italy (37, accounting for 3.03%), Netherlands and Portugal (both 27, accounting for 2.21% respectively). The number of publications in ten countries, including Korea, Denmark, Argentina, Norway, France, Poland, Sweden, Belgium, Switzerland and Australia, ranges from 14 to 25, while other countries are less than 14. The number of publications published by China and the United States constitutes a huge part of the overall documents (388, accounting for 31.75%). Among the countries with more than 30 publications, except for China and Brazil, the rest are developed countries. Single Country Publications (SCPs) and Multiple Country Publications (MCPs) can reflect the internal cooperation of countries in kinship analysis (Figure 4). China has the highest number of SCPs, while the United States has the second highest number of SCPs.

**TABLE 1:** Top 20 countries in the field of kinship analysis with the highest number of articles.

Country	Publications	Publication percentage(%)	SCP	MCP	MCP ratio	Citations	Average publication citations
China	213	17.43	188	25	0.117	2099	9.90
United States of America	175	14.32	130	45	0.257	7692	44.00
Germany	89	7.28	71	18	0.202	2563	28.80
Japan	57	4.66	50	7	0.123	479	8.40
Brazil	53	4.34	42	11	0.208	810	15.30
Spain	48	3.93	25	23	0.479	1091	22.70
United Kingdom	47	3.85	31	16	0.34	3145	66.90
Italy	37	3.03	26	11	0.297	395	10.70
Netherlands	27	2.21	16	11	0.407	2528	93.60
Portugal	27	2.21	16	11	0.407	466	17.30
Korea	25	2.05	23	2	0.08	281	11.20
Denmark	24	1.96	18	6	0.25	855	35.60
Argentina	18	1.47	8	10	0.556	194	10.80
Norway	18	1.47	4	14	0.778	428	23.80
France	17	1.39	8	9	0.529	763	44.90
Poland	17	1.39	14	3	0.176	163	9.60
Sweden	17	1.39	9	8	0.471	302	17.80
Belgium	15	1.23	9	6	0.4	242	16.10
Switzerland	15	1.23	12	3	0.2	326	21.70
Australia	14	1.15	11	3	0.214	139	9.90

**FIGURE 5:**
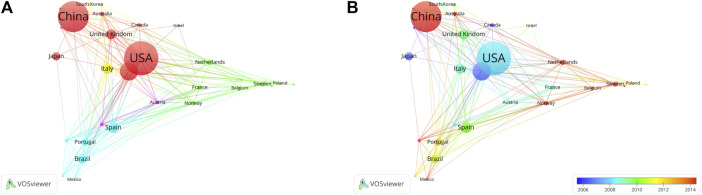
Co-occurrence analysis of countries. **(A)** Collaborative relationships among various countries in kinship analysis were visualized using VOSviewer. The visualization displays a network of diverse countries. Each node corresponds to an individual country, and the size of each circle is determined by the quantity of publications. The connecting lines symbolize collaborations among countries. The lines connecting items depict links, and the distance between two items roughly indicates their level of relatedness. Various colors denote distinct items. **(B)** Collaborative relationships among various countries in the field of kinship analysis were visualized using VOSviewer. Illustrate the distribution of countries based on the average timing of their contributions. Green and blue circles represent earlier publications, while yellow circles denote more recent ones.

The United States has the most citations, with 7,692 (an average of 44.00 citations per article) (Table 1). The United Kingdom followed with 3,145 citations (an average of 66.90), Germany with 2,563 citations (an average of 28.80), Netherlands with 2,528 citations (an average of 93.60), and China with 2,099 citations (an average of 9.90). In terms of average article citations, Netherlands, the United Kingdom and France rank in the top three. Using VOSviewer, we screened countries and regions with more than 10 published publications, and 30 out of 95 countries and regions met the criteria (Figure 5A). The graph illustrating the co-occurrence relations among countries presents Five distinct clusters: Cluster 1: Australia, Canada, China, Finland, Germany, Israel, Japan, Pakistan, United Kingdom, United States. Cluster 2: Belgium, France, Netherlands, Norway, Poland, Russia, Sweden, Switzerland, Turkey. Cluster 3: Argentina, Brazil, Colombia, Mexico Portugal, Spain. Cluster 4: India, Italy, South Korea. Cluster 5: Austria, Denmark. The research in the field of kinship analysis in the United States started earlier, while that in China started late (Figure 5B).

**FIGURE 6:**
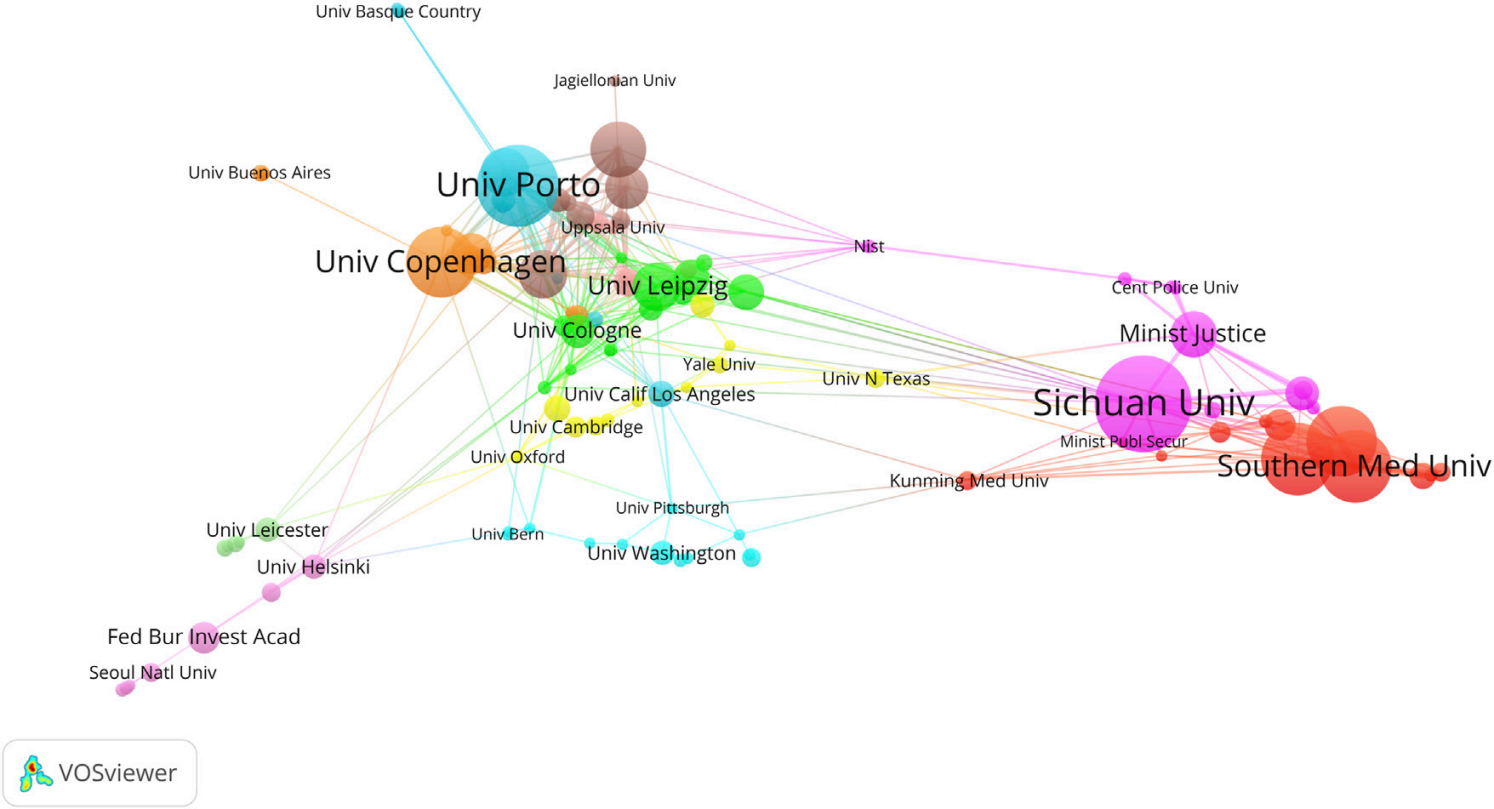
The co-authorship network visualization map of institutions in kinship analysis were visualized using VOSviewer. The visualization depicts a network of diverse organizations. Each node signifies an individual organization, and the size of each circle corresponds to the quantity of publications. The connecting lines symbolize collaborations among organizations. The lines connecting items depict links, and the distance between two items roughly indicates their level of relatedness. Nodes of the same color signify membership in the same cluster.

The Sichuan University ranked first with 40 articles, followed by the University of Porto (34), the Southern Medical University (30), and the Sun Yat-Sen University (30) (Figure 6; Table 2). The University of Cologne has the highest mean article citations, with 14 articles and an average of 77.93 citations per article, followed by the University of Leipzig (20 articles, average 63.25 citations) and Technische Universität Dresden (15 articles, average 53.00 citations). Total Link Strength (TLS) can reflect collaborative research between institutions. The Sichuan University has the highest TLS score with 53 points. Norwegian University of Life Sciences and the University of Proto ranked second and third with 52 and 49 points respectively. The TLS scores, combined with the national cooperation network map (Figure 6), indicated that the Sichuan University, University of Porto, the Southern Medical University, the Sun Yat-Sen University, Southern Medical University, and University of Copenhagen play significant roles as research partners for multiple institutions.

**TABLE 2:** Top 20 organizations in the field of kinship analysis with the highest number of documents. The total link strength in the table indicates the total strength of the co-authorship links of a given organization with other organizations.

Organization	Country	Publications	Citations	Average publication citations	Total link strength
Sichuan University	China	40	442	11.05	53
University of Porto	Portugal	34	755	22.21	49
Southern Medical University	China	30	225	7.50	48
Sun Yat-Sen University	China	30	346	11.53	22
University of Copenhagen	Sweden	29	1166	40.21	31
Xi’an Jiaotong University	China	29	295	10.17	33
National Board of Forensic Medicine	Denmark	23	552	24.00	48
Norwegian University of Life Sciences	Norway	20	534	26.70	52
University of Leipzig	Germany	20	1265	63.25	43
University of Santiago de Compostela	Spain	20	1020	51.00	26
Institute of Forensic Science Ministry of Justice PRC	China	19	271	14.26	28
Linköping University	Sweden	18	463	25.72	43
Katholieke Universiteit Leuven	Belgium	17	377	22.18	11
Technische Universität Dresden	Germany	15	795	53.00	28
Kiel University	Germany	15	499	33.27	21
Fudan University	China	14	121	8.64	14
University of Cologne	Germany	14	1091	77.93	43
Chinese Academy of Sciences	China	13	280	21.54	16
Federal Bureau of Investigation Academy	United States	13	405	31.15	3
Netherlands Forensic Institute	Netherland	12	332	27.67	16

### 3.3 Most productive authors

**FIGURE 7:**
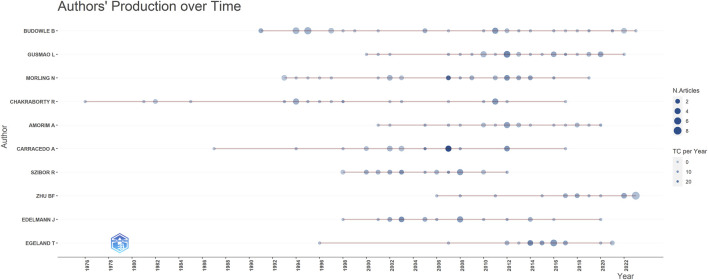
Contribution of top 10 authors over different years (red lines). The size of dots indicates the number of publications over different years, and the color of dots (light to dark) indicates total citations (TC) per year.

The most prolific authors in terms of articles were Budowle B (40 articles, 968 citations), Gusmao L (31 articles, 624 citations), and Morling N (29 articles, 1,186 citations) (Table 3). However, the number of articles authored by individuals did not necessarily correlate with the number of citations received. For example, Kayser M who authored fewer articles (15, ranking 11th), received 2,267 citations, whereas Budowle B, with the highest number of articles (40), received only 968 citations. To address this disparity, we introduce evaluation metrics such as the h-index, g-index, and m-index. Authors with the highest h-index scores were Budowle B (h-index of 18), Morling N (h-index of 17), and Edelmann J (h-index of 16). Budowle B (g-index of 30), Morling N (g-index of 29), Chakraborty R (g-index of 24) and Gusmao L (g-index of 24) achieved the top four g-index scores. In terms of the m-index, Pinto N had the highest score (m-index of 0.667), followed by MHD (m-index of 0.643), Edelmann J (m-index of 0.593), Szibor R (m-index of 0.593). Budowle B, ranked first in the number of published articles, published the largest number of articles in 2011 (Figure 7). Gusmao L, who ranked second, began to explore kinship analysis in 2000 and made the most significant contribution in 2010. Morling N ranked third, having started their research in the field since 1993 and published the most articles in 2002, 2012.

**TABLE 3:** Top 20 authors in the field of kinship identification with the highest number of H-index. TC = Total citations; NP = Number of productions; PY start = Publication years start.

Author	H-index	G-index	M-Index	TC	NP	PY start	TC per year
Budowle B	18	30	0.529	968	40	1991	29.33
Morling N	17	29	0.531	1186	29	1993	38.26
Edelmann J	16	20	0.593	934	20	1998	35.92
Szibor R	16	21	0.593	1178	21	1998	45.31
Chakraborty R	15	24	0.306	925	24	1976	19.27
Carracedo A	14	21	0.368	1286	21	1987	34.76
Gusmao L	14	24	0.56	624	31	2000	26
Hering S	14	18	0.56	905	18	2000	37.71
Schneider PM	14	16	0.424	987	16	1992	30.84
Amorim A	13	21	0.542	441	21	2001	19.17
Kayser M	12	15	0.444	2267	15	1998	87.19
Egeland T	11	20	0.379	533	20	1996	19.04
Parson W	11	15	0.393	489	15	1997	18.11
Krawczak M	10	18	0.303	664	18	1992	20.75
Pinto N	10	15	0.667	244	15	2010	17.43
Sajantila A	10	11	0.294	812	11	1991	24.61
Augustin C	9	12	0.474	361	12	2006	20.06
Brinkmann B	9	11	0.176	363	11	1974	7.26
Ge JY	9	16	0.5	303	16	2007	17.82
Larmuseau MHD	9	12	0.643	338	12	2011	26

### 3.4 Analysis of high-yielding journals

Table 4 provides a list of the most prominent journals that have published research articles on kinship identification. *Forensic Science International-Genetics* (249 articles, 5,011 citations) is the most popular journal for kinship analysis in terms of publications. *International Journal of Legal Medicine* (228 articles, 3,766 citations) ranks second, followed by *Forensic Science International* (154 articles, 3,538 citations), *Journal of Forensic Sciences* (93 articles, 1,576 citations), *Legal Medicine* (49 articles, 326 citations), and *American Journal of Human Genetics* (41 articles, 2,364 citations). In terms of citations, the ranking is as follows: *Forensic Science International-Genetics* holds the top position, followed by *International Journal of Legal Medicine*, *Forensic Science International*, *American Journal of Human Genetics*, and *Journal of Forensic Sciences*.

**TABLE 4:** Top 20 sources in the field of kinship identification with the highest number of H-index. TC = Total citations; NP = Number of productions; PY start = Publication years start.

Sources	H-index	G-index	M-Index	TC	NP	PY start	TC per year
*Forensic Science International-Genetics*	35	55	1.944	5011	249	2007	294.76
*Forensic Science International*	31	52	0.721	3538	154	1982	84.24
*International Journal of Legal Medicine*	29	47	0.853	3766	228	1991	114.12
*American Journal of Human Genetics*	21	41	0.404	2364	41	1973	46.35
*Journal Of Forensic Sciences*	21	37	0.488	1576	93	1982	37.52
*Human Biology*	12	21	0.353	489	21	1991	14.82
*Human Genetics*	11	16	0.224	722	16	1976	15.04
*Legal Medicine*	10	14	0.667	326	49	2010	23.29
*European Journal of Human Genetics*	9	13	0.346	270	13	1999	10.8
*Human Heredity*	9	19	0.205	391	21	1981	9.09
*Genetic Epidemiology*	8	14	0.308	453	14	1999	18.12
*Heredity*	8	8	0.421	499	8	2006	27.72
*Frontiers In Genetics*	6	11	0.545	147	23	2014	14.7
*Gene*	6	9	0.6	94	10	2015	10.44
*Genes*	6	9	1	113	25	2019	22.6
*Genetics*	6	7	0.214	1171	7	1997	43.37
*PLOS Genetics*	6	8	0.545	773	8	2014	77.3
*Behavior Genetics*	5	8	0.161	392	8	1994	13.07
*Genome Research*	5	5	0.167	663	5	1995	22.86
*Molecular Genetics and Genomic Medicine*	5	5	0.833	41	5	2019	8.2

**FIGURE 8:**
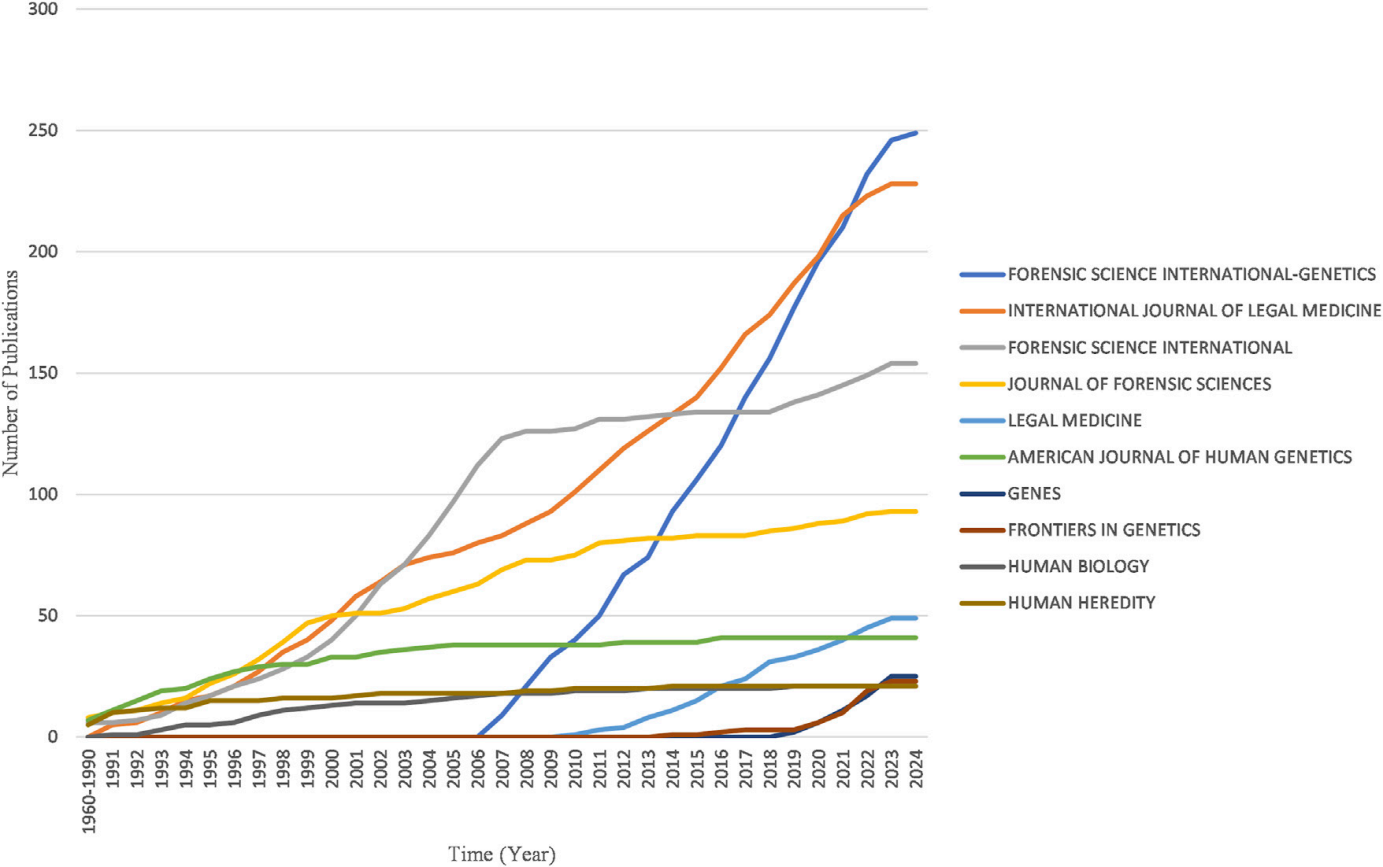
Yearly publication growth trend of top 10 sources in the field of kinship identification with the highest number of documents.

Figure 8 reveals the four major journals with the fastest growth in publications over the past decade: *Forensic Science International-Genetics, International Journal of Legal Medicine, Forensic Science International and Journal of Forensic Sciences.*

### 3.5 Analysis of number of citations

The publications were ranked by number of citations. Among the top 20 cited articles (Table 5), the most cited article is “Van Oven M, 2009, Hum Mutat” with 1,361 citations. In this study (van Oven and Kayser, 2009), the author constructed an updated comprehensive phylogeny of global human mitochondrial DNA (mtDNA) variation, based on both coding and control region mutations. “Wang JL, 2004, Genetics” ranks second with 777 citations, followed by “Liu XL, 2016, PLOS Genet” and “Queller DC, 1993, Trends Ecol Evol” with 627 and 613 citations, respectively. Only four articles have more than 500 citations, while the subsequent 15 articles all had more than 200 citations.

**TABLE 5:** Top 20 articles in the field of kinship identification with the highest number of citations. TC = Total citations.

Article	Total citations	TC per year
Van Oven M, 2009, Hum Mutat	1361	85.06
Wang JL, 2004, Genetics	777	37.00
Liu XL, 2016, PLOS Genet	627	69.67
Queller DC, 1993, Trends Ecol Evol	613	19.16
Snook RR, 2005, Trends Ecol Evol	485	24.25
Blouin MS, 2003, Trends Ecol Evol	483	21.95
Balding DJ, 1994, Forensic Sci Int	333	10.74
Jobling MA, 1997, Int J Legal Med	292	10.43
Sobel E, 2002, Am J Hum Genet	278	12.09
Sobrino B, 2005, Forensic Sci Int	273	13.65
Oliveira EJ, 2006, Genet Mol Biol	248	13.05
Prinz M, 2007, Forensic Sci Int-Gen	228	12.67
Szibor R, 2003, Int J Legal Med	224	10.18
Posthuma D, 2000, Behav Genet	221	8.84
Gymrek M, 2012, Genome Res	211	16.23
Conomos MP, 2015, Genet Epidemiol	210	21.00
Abecasis GR, 2005, Am J Hum Genet	207	10.35
Desmarais D, 1998, J Forensic Sci	207	7.67
Conomos MP, 2016, Am J Hum Genet	206	22.89
Conomos MP, 2016, Am J Hum Genet-A	198	22.00

### 3.6 Co-occurrence keywords with their changing trend

**FIGURE 9:**
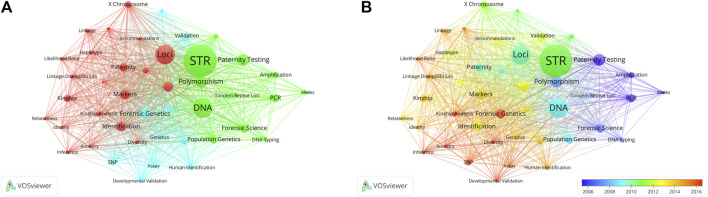
Co-occurrence analysis of countries. **(A)** The co-occurrence network of keywords is depicted. The lines connecting nodes indicate co-occurrence among distinct keywords. Distinct colors in the figure signify clusters, each comprising closely related nodes or items. Each network item is assigned to a single cluster, with an item’s color determined by its cluster membership. Connecting lines between items represent links, and the distance between two items roughly indicates their relatedness. **(B)** Overlay Visualization illustrating keywords. Display the keywords based on their average timing of occurrence. Green and blue circles represent earlier publications, while yellow circles denote more recent ones.

Co-occurrence keywords that appeared at least 31 times were taken into account (Figure 9A). Of the 3,614 results, 50 met the threshold. Among these co-occurrence keywords, “DNA” was the word with the highest frequency (217 times) in all articles, followed by “Loci” (180 times), “Paternity testing” (127 times), “Population” (109 times), “Markers” (104 times) and “Identification” (103 times). The co-occurring terms in the timeline (Figure 9B) have undergone a gradual shift from “Paternity testing”, “PCR” and “Polymorphism” to “STR”, “DNA” and “Markers”. Subsequently, they further evolved into “Kinship analysis”, “SNP” and “Inference".

### 3.7 Trend topic

**FIGURE 10:**
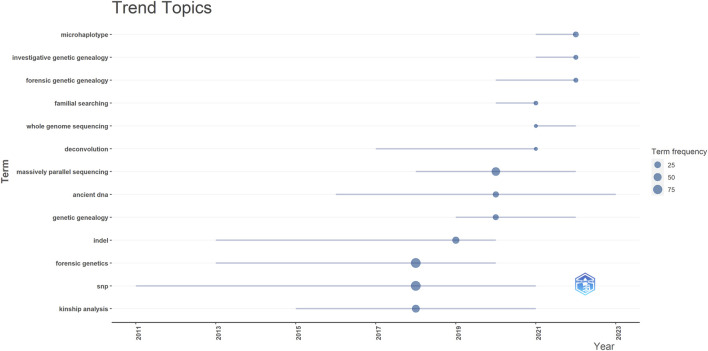
Trend topic from 2018 to 2023. InDel = Insertion-Deletion, SNP = Single Nucleotide Polymorphism.

The topic trends in this field are examined by analyzing keywords using Biblioshiny (RStudio) from 2018 to 2023 (Figure 10). In the initial phase, research in this field was characterized by prominent keywords such as “Kinship analysis” (44 occurrences), “SNP” (87 occurrences), and “Forensic Genetics” (91 occurrences). Notably, among all the keywords, “Forensic Genetics” emerged with the highest frequency. In recent years, the research focus and trend have shifted towards microhaplotypes, forensic genetic genealogy and massively parallel sequencing.

## 4 Discussion

Kinship analysis plays a pivotal role in numerous fields, owing to its ability to uncover relationships among individuals and fathom profound significance within social structures, biological relationships, and historical contexts. In genetic research, kinship analysis provides invaluable insights into inherited diseases, population genetics, and evolutionary studies. By studying the genetic relatedness between individuals, scientists have discovered genetic markers for various diseases and traced the migration patterns of ancient human populations (Klein et al., 2005; Kruglyak, 2005; Chen and Nedoluzhko, 2023). Additionally, kinship inference proves pivotal in forensic genetic genealogy, not only facilitating the exploration of ancestral origins (Mateen et al., 2021) and the tracing of family trees (Willson et al., 2022), but also aiding public security in solving criminal cases (Greytak et al., 2019). For instance, genealogy websites and DNA testing services have fostered people to connect with long-lost relatives and gain a deeper understanding of their roots (Khan and Mittelman, 2018). Moreover, in legal matters, kinship analysis assists in determining legal rights, inheritance, and the resolution of disputed relationships. Courts often rely on kinship identification to establish biological or legal relationships in disputes over inheritance or child custody.

### 4.1 Chronological distribution of publications

In this bibliometric analysis, we sieved through 1,222 documents related to kinship analysis from the Web of Science Core Collection database. Publications on kinship analysis have grown steadily since 2010, nearly three times by 2023. This growth is driven by the ever-expanding interest in understanding human relationships and genetics. Researchers and scholars have actively contributed to the field, catalyzing a notable increase in publication volume. The availability of advanced technologies and improved research methods have also supported the rise of publications. Since the establishment of the first STR database in 1995 (Amankwaa and McCartney, 2018), an average annual publication output of roughly 20 has been consistently observed. In 2010, The 1000 Genomes Project Consortium published an article in *Nature* that included information on 15 million SNPs and one million InDels, served as a watershed moment (Consortium, 2010). Subsequently, more than 30 publications have been emerged annually, except 2013. In 2018, the Golden State killer was arrested through forensic genetic genealogy (Phillips, 2018). This new kinship inference method has attracted widespread attention. Since then, the publication output has surpassed 50 papers every year. In terms of citations, it is noteworthy that high citation frequencies were observed in 1993, 2005, and 2009. The elevated citation rate in 1993 can be attributed to the gradual exploration of the utility of microsatellite markers in forensic genetics, characterized by their high variability among individuals and their efficacy in discerning relatedness between samples (Queller et al., 1993). The peak citation rate in 1995 was due to a novel genetic marker called SNP, which demonstrated considerable potential for complex kinship analysis and offered advantages over STR (Sobrino et al., 2005). The significant increase in citation rates in 2009 was primarily attributed to the publication of a comprehensive phylogeny on global human mtDNA variation (van Oven and Kayser, 2009) as well as research conducted on the human Y chromosome (Goedbloed et al., 2009; King and Jobling, 2009).

### 4.2 The contributions of countries and organizations

We first analyze the contributions of countries, organizations, authors and journals in kinship analysis research. China and the United States have emerged as the leading contributors in this field, accounting for a substantial 388 publications, which represents over 30 percent of the total. Conversely, contributions from other nations have remained modest, with publication counts below 100. Furthermore, when considering average citation rates of scholarly articles authored by researchers from the United States, they rank fourth globally, trailing only behind the Netherlands, United Kingdom, and France. This finding underscores the significant influence wielded by the United States in this domain. This dominant position of the United States in the field of kinship analysis can be ascribed to various factors. First, the United States has the highest MCP, indicating that American scholars have closer intercommunication with peers across nations and are more likely to produce high-quality results. Additionally, it suggests that American scholars benefit from extensive collaborations with their international counterparts, leading to a profound impact on scholarly work. What’s more, this ascendency is also attributable to the great economic and scientific research strength of the United States (Lei et al., 2019; Chen Z. et al., 2023).

Despite China having the largest number of publications in kinship analysis, its average citation ranking among the top 20 countries is only 17th. The average citations are often served as a barometer of a research work’s influence and value within its field. The lower average citation might be attributed to the shorter publication time of Chinese papers (Figure 5), which also implies that Chinese scholars need to prioritize the quality of their research outcomes over sheer quantity. Nevertheless, it is noteworthy that China, as a developing country, has made substantial strides in kinship analysis research with the largest number of publications, holding promising prospects for the future. Domestic collaboration trends among nations underscore the need for increased international exchange in the field of kinship analysis. International collaboration fosters a broader perspective and a thorough understanding of kinship analysis.

Sichuan University in China has the largest number of published articles. The University of Leipzig and the University of Cologne in Germany exhibit the highest total citation count and average citation, respectively, indicating that Germany’s notable influence in this field. Among the top 20 institutions, seven hail from China, four from Germany, two from Sweden, and the remaining seven from developed countries. Research results are closely linked to financial investment, personnel training, research culture, and international collaboration. Developed nations possess greater resources and talent for conducting kinship inference research. Prestigious universities with rich academic achievements are more likely to garner increased support, attract superior talents, secure ample funding for scientific research, and cultivate an environment conducive to innovation and exploration. Moreover, they also enjoy more opportunities for global exchange.

### 4.3 The impact of authors and journals

What’s more, Professor Budowle B from the University of North Texas Health Science Center in the United States published the most articles, renowned for his expertise in forensic science, specializing in the DNA identification of missing individuals in mass disasters (Budowle et al., 2005b), as well as the development and application of sequencing technology (Seo et al., 2013; Warshauer et al., 2013; Zeng et al., 2015), forensic microbiology (Schmedes et al., 2016; Schmedes et al., 2017), and genetic marker loci research (Budowle et al., 2005a; Larue et al., 2012). Meanwhile, Kayser M from Erasmus MC University Medical Center Rotterdam in Netherlands commands the highest citation count and average citation rate in his field, largely attributable to his pioneering work on the global human mtDNA variation (van Oven and Kayser, 2009). KENNETT D from University College London, who has the highest m-index and works on FGG, described to us the process by which dense SNP data are used to infer distant relationships (Kling et al., 2021). *Forensic Science International-Genetics*, *Forensic Science International*, and *International Journal of Legal Medicine* are the top three journals by the number of publications in this study. Furthermore, *Forensic Science International-Genetics* boasts the highest citation rate within this field. A bibliometric analysis of forensic genetics also found that *Forensic Science International-Genetics*’ preeminence, with the highest number of articles and citations in forensic genetics (Stasi et al., 2023). For scholars conducting kinship analysis research and aiming to publish in high-impact journals, *Forensic Science International-Genetics* could be considered a favorable option. This journal provides an excellent platform for researchers in the field of kinship analysis to show their work and contribute to the advancement of forensic genetics.

### 4.4 Genetic markers contribute to complex kinship analysis

With the rapid development of society, kinship analysis of the parent-child relationship has been unable to meet the needs of disaster victim identification and criminal investigations. Complex kinship encompasses relationships such as grandparent-grandchild, uncle/aunt-nephew/niece, full sibling, half-sibling, and first or second cousins. The first cousins share a grandparent (2 generations) and the second cousins share a great-grandparent (3 generations). Currently, there are some genetic markers employed in forensic DNA analysis to address complex kinship analysis, such as autosomal STRs, Y-chromosomal STRs, X-chromosomal STRs, mtDNA, SNPs, InDels and MHs.

Autosomal STRs account for about 5% of the human genome, of which about 50% have genetic polymorphisms, mainly distributed in non-coding regions, and are suitable for most complex kinship analysis. However, conventional STR detection methods utilizing CE technology typically only amplify less than 50 STR loci (Martín et al., 2014; Wang et al., 2015; Song et al., 2023), and is difficult to obtain complete STR profiles for trace DNA less than 100 pg (Xu et al., 2022). This restricted range of STR loci greatly and hampered its applicability in intricate kinship analysis. To overcome this limitation, Cong et al. capitalized on the high-throughput capability of the NGS method, and enabled simultaneous sequencing of numerous genomic regions in a single reaction (Børsting and Morling, 2015). They successfully developed an NGS-STR typing system including 42 autosomal STR loci and an amelogenin marker, which showed remarkable efficacy for 2nd-degree kinship analysis (Liu et al., 2020).

The sex STR markers specifically target regions on the Y chromosome for males and the X chromosome for females. Y-STR haplotype analysis as a prevalent tool for paternal kinship testing in historical cases, missing persons and disaster victim identification involving males. In contrast to autosomal STR, Y-STR profiling can trace distant relatives and circumvent the potential sharing of autosomal alleles between victim and perpetrator in sexual assault cases (Kayser, 2017). Moreover, rapidly mutating (RM) Y-STRs have been reported to be able to successfully differentiate between close and distant male relatives (Ballantyne et al., 2012; Ralf et al., 2020; Ralf et al., 2021; Wang F. et al., 2022). Due to the higher mutation rate of RM Y-STRs in comparison to standard Y-STRs, they significantly enhance the differentiation among male relatives within the same paternal lineage (Wang F. et al., 2022). Nevertheless, Y-STR haplotypes have a higher variability compared to single autosomal STR loci and therefore it is imperative for the Y-STR haplotype database to possess a larger scale than the autosomal STR allele database in order to ensure reliability (Kayser, 2017), which implies an additional investment in both time and financial resources. In complex kinship analysis, such as full-sib girls or half-sib girls, the use of X-STR is particularly important because it has a higher ability to exclude or identify than autosomal STR (Kling et al., 2015). The development of X-STR has also been shown to solve complex kinship in the case that X-chromosomal lineages can be taken under investigation (Becker et al., 2008).

Moreover, by using autosomal STRs alongside sex STRs in kinship analysis, researchers can obtain a more accurate assessment of biological relatedness. This dual-marker strategy helps overcome limitations that may arise when relying solely on either autosomal or sex-linked genetic data. For instance, while autosomal STRs offer broader coverage across all chromosomes and can be used to analyze relationships between any two individuals regardless of their gender, they may not always provide conclusive results due to factors like mutations or shared ancestry within populations (Amorim and Pereira, 2005). In a recent study on skeletons of Romanized indigenous people from the 5th to 6th century, researchers utilized autosomal STR typing and the PowerPlex Y23 kit for Y-STR typing, confirming that four skeletons were members of the same family (a father, two daughters, and a son) (Pajnič et al., 2023). MtDNA is particularly advantageous in cases with limited nuclear DNA or when confirmation of maternal lineage (van Oven and Kayser, 2009; Syndercombe Court, 2021). Its efficacy in the analysis of bones, teeth, and hair makes it a common choice for ancient DNA research and disaster victim identification triage (Kurosaki et al., 1993; Syndercombe Court, 2021). Despite its advantages, mtDNA analysis encounters difficulties when dealing with ancient or degraded samples, as contamination, amplification verification, and interference from other genomic regions can pose issues (Loreille et al., 2018; Syndercombe Court, 2021).

Compared with STR, SNPs, InDels and MHs are novel genetic markers and well suited for kinship analysis, characterized by a lower mutation rate, shorter amplicon size, high stability and the absence of a stutter peak (Børsting et al., 2012; Wei et al., 2014; de la Puente et al., 2020; Bai et al., 2022; Yu et al., 2022; Yuan et al., 2024). The determination of the number and lengths of identity by descent segments using high-density SNP or whole-genome sequence data is a fundamental principle of FGG (Huff et al., 2011; Ertürk et al., 2022). However, binary markers like SNPs and InDels are comparatively lower than that of STR in polymorphism, necessitating a greater number of binary genetic markers to achieve an equivalent information content as observed with STR markers (Amorim and Pereira, 2005; Wei et al., 2014; Yuan et al., 2024). InDels capitalize on the benefits of SNPs and STRs, as they can be analyzed through PCR-to-CE typing approach (Liu et al., 2017; Oldoni and Podini, 2019). MHs are genetic markers that are generally less than 300 bp and consist of a small cluster of closely linked SNPs (de la Puente et al., 2020; Bai et al., 2022; Yu et al., 2022). MH loci are single-copy and have multiple SNPs, providing more information per locus than a single SNP, but still with lower polymorphism compared to STR loci (Oldoni and Podini, 2019).

### 4.5 The development and challenges brought by technological advancement

Sequencing technologies, especially NGS and third-generation sequencing (TGS), have become a hot research topic and trend in recent years and have had a great impact on kinship analysis. NGS technology can be utilized in the identification of SNPs, InDels, mtDNA, and STRs (Ballard et al., 2020; Feng et al., 2024). Compared to traditional CE analysis, NGS offers the following advantages: 1) It can simultaneously detect a large number of STR loci and has the ability to distinguish alleles with similar lengths or digital read count (Yang et al., 2014); 2) It is suitable for samples with low DNA content or degradation (Scheible et al., 2014); 3) NGS analysis can determine the full sequence of PCR products, including the STR repeat region and flanking regions (Ballard et al., 2020), allowing more variant information to be observed (Gettings et al., 2017; Phillips et al., 2018; Davenport et al., 2023) and facilitating the differentiation of mixed DNA (Phillips et al., 2007; Devesse et al., 2018). The high-throughput, fast and low cost NGS technology has brought a new revolution in forensic science. The detection of DNA sequence polymorphism has been enhanced, and the exploration of novel genetic markers is steadily advancing. The advantages of MHs in the fields of mixture deconvolution (Oldoni et al., 2020; Tao et al., 2022; Yu et al., 2022), biogeographic ancestry inference (de la Puente et al., 2020; Tao et al., 2022), complex kinship analysis (Wen et al., 2022; Xue et al., 2023) and personal identification (Pu et al., 2017) have been extensively investigated. Based on NGS, FGG has played a significant role in identifying unknown remains (Bertoglio et al., 2020), inferring distant relatives (Kling et al., 2021), and solving cold cases (Phillips, 2018).

Since Joseph James DeAngleo was successfully identified as the prime suspect in the Golden State killer case in 2018 (Phillips, 2018), forensic genetic genealogy has garnered widespread attention. The application of FGG has been reported to generate investigative leads in unresolved cold cases, and hundreds of cases have been solved using FGG technology (Murphy, 2018; Ram et al., 2018; Glynn, 2022). FGG employs a set of high-density SNPs profiles by microarray or whole-genome sequencing (WGS) to genotype biological samples or determine relatedness (Ertürk et al., 2022). SNP profiles are provided by high-density SNP profile databases such as GEDmatch, FamilyTreeDNA and DNASolves (Glynn, 2022), with data collected via direct-to-consumer (DTC) genetic testing (Majumder et al., 2021). Traditional kinship identification is mainly based on STR markers and analyzed by identical by state (IBS) or likelihood ratio (LR). In contrast, FGG mainly relies on whole genome sequencing or high-density chip autosomal SNP typing and is analyzed by method-of-moment (MoM) or identical by descent (IBD) fragments (Ge and Budowle, 2021; Kling et al., 2021). FGG presents advantages over traditional kinship analysis methods. FGG can be used for kinship identification at the fifth-degree and beyond, whereas traditional kinship identification cannot (Glynn, 2022). While traditional complex kinship analysis needs to increase the number of loci, FGG only needs to be tested once through WGS. However, for relationships beyond third-generation cousins (seventh-degree relationships) or more distant, individuals may not share any IBD fragments (Edge and Coop, 2020). Moreover, in certain cases, numerous relatives may present matches, demanding substantial resources for screening (Court, 2018). Additionally, it is controversial whether the police have the right to access the data and use it in the investigation of cases (Ram and Roberts, 2019), but Sweden has reported successful use of FGG in case detection work (Tillmar et al., 2021) and the United Kingdom (Samuel and Kennett, 2020), Australia (Scudder et al., 2020) are contemplating future utilization of this technology.

The application of TGS technology in forensic genetics is burgeoning, providing a new method for real-time detection of longer markers through its single molecule sequencing and long-read techniques (Athanasopoulou et al., 2021; White and Hesselberth, 2022). Hou et al. (Wang Z. et al., 2022) employed the QNome, a nanopore genome sequencer developed by Qitan Technology, to genotype 15 MHs from 70 single-contributor samples, achieving an accuracy of 99.83%. This highlights the potential of the nanopore sequencing method in forensic analysis of MH markers. Additionally, large-scale genome projects have profound implications for kinship analysis, as they offer a vast amount of genetic data that can be harnessed by forensic experts. Through the examination of this data, forensic experts are able to identify and investigate new genetic markers relevant to forensic, thereby enhancing their capabilities in complex kinship analysis (Kureshi et al., 2020; Phillips et al., 2020; Frontanilla et al., 2022; Xue et al., 2022).

### 4.6 Challenges and future directions

The extraction of trace DNA and the analysis of mixed DNA remain ongoing challenges in the field (Supplementary Table S1). It has been observed that obtaining complete STR profiles becomes challenging when working with DNA samples containing less than 100 pg (Xavier et al., 2020; Xu et al., 2022). This limitation in sample size could potentially compromise the accuracy and reliability of kinship analysis. Analyzing ancient DNA approaches and suggesting systematic research on DNA extraction methods could improve the quality and quantity of DNA (Hofreiter et al., 2021). Furthermore, crime scenes frequently present investigators with mixed DNA samples, which consist of genetic material from multiple individuals. These mixed samples pose additional challenges during analysis due to a higher probability of drop-out or drop-in combined with stutter peak (Gill et al., 2012; Bai et al., 2022). To overcome these hurdles, MHs have been extensively studied by scholars due to their combined advantages of STR and SNP markers, such as low mutation rate, high polymorphism, short length, and absence of stutter peaks (Kidd et al., 2013; Oldoni et al., 2019; Bai et al., 2022; Wen et al., 2022). Bai et al. developed a large panel consisting of 185 MHs to analyze degraded and/or mixed DNA samples demonstrating its utility in conducting parentage, full sibling, and second-degree relative testing, but improvements are necessary to infer more distant relatives (third-degree relatives) (Bai et al., 2022). Furthermore, the application of single cell sequencing (SCS) technology, recognized as one of the top ten scientific breakthrough technologies in 2018 along with FGG by *Science* (Chen L. et al., 2023), warrants attention in kinship analysis. While FGG has been a research focus in forensic genetics, SCS has received less attention despite its promising potential. SCS is widely used in developmental biology, the generation of human cell maps, and cancer research (Tirosh et al., 2016; Venteicher et al., 2017). However, it is theoretically feasible to achieve complete sequencing with only a single cell, offering potential solutions to forensic challenges arising from trace DNA (Zong et al., 2012; Diepenbroek et al., 2021). What’s more, single-cell separation technology facilitates the isolation of individual cells from mixed samples, thereby eliminating the mixture and improving deconvolution (Farash et al., 2018; Diepenbroek et al., 2021). This approach, when applied to complex familial mixtures, can prevent the erroneous inclusion of non-donor relatives (Huffman and Ballantyne, 2022). Nevertheless, SCS also encounters challenges such as limited automation, reduced accuracy, and restricted applicability of DNA typing results in databases (Huffman and Ballantyne, 2023).

In addition to the aforementioned challenges, the realm of complex kinship analysis demands attention as well. Complex kinship relationships involve grandparent-grandchild, uncle/aunt-nephew/niece, full sibling, half sibling, and first or second cousins. Identifying these relationships accurately can be particularly difficult due to their intricate nature. To enhance the accuracy of identification in complex kinship analysis, forensics may add STR, SNP and InDel genetic markers or adopt novel genetic markers (Zhang et al., 2022). The emergence of FGG has also significantly promoted distant relative inference (Glynn, 2022). However, even with advancements in technology and methods, kinship analysis of identical twins still remains notably challenging (Yuan et al., 2020). Currently, it is promising to identify monozygotic twins by ultra-deep next-generation sequencing to identify rare mutations (Weber-Lehmann et al., 2014), Various CpG sites (Mill et al., 2006; van Dongen et al., 2021) and microbial communities (Fierer et al., 2010; Martinez et al., 2013). In conclusion, the future research trend will involve the identification of novel genetic markers and the development of advanced analytical techniques. Challenges that still exist in this field include accurately and rapidly analyzing complex kinship, as well as successfully typing degraded DNA or mixed samples.

## 5 Conclusion

This study primarily focuses on the global trends and development of kinship analysis. From an overall perspective, research in the field of kinship analysis has gradually gained attention, with an increasing number of papers published over the years. In terms of countries, this relevant research is mainly driven by developed and developing nations such as China, the United States, and Germany. Looking ahead, there is a desire to enhance international exchanges and involve more countries in kinship analysis research. Simultaneously, research in the field of kinship analysis concentrates on the identification of novel genetic markers and development of advanced analytical techniques. However, numerous challenges still exist within this domain.

## Data Availability

The raw data supporting the conclusion of this article will be made available by the authors, without undue reservation.

## References

[B1] Alterauge A. Lösch S. Sulzer A. Gysi M. Haas C. (2021). Beyond simple kinship and identification: aDNA analyses from a 17th-19th century crypt in Germany. Forensic Sci. Int. Genet. 53, 102498. 10.1016/j.fsigen.2021.102498 33872864

[B2] Amankwaa A. O. McCartney C. (2018). The UK National DNA database: implementation of the protection of freedoms act 2012. Forensic Sci. Int. 284, 117–128. 10.1016/j.forsciint.2017.12.041 29367171

[B3] Amorim A. Pereira L. (2005). Pros and cons in the use of SNPs in forensic kinship investigation: a comparative analysis with STRs. Forensic Sci. Int. 150 (1), 17–21. 10.1016/j.forsciint.2004.06.018 15837005

[B4] Athanasopoulou K. Boti M. A. Adamopoulos P. G. Skourou P. C. Scorilas A. (2021). Third-generation sequencing: the spearhead towards the radical transformation of modern genomics. Life (Basel) 12 (1), 30. 10.3390/life12010030 35054423 PMC8780579

[B5] Bai Z. Zhang N. Liu J. Ding H. Zhang Y. Wang T. (2022). Identification of missing persons through kinship analysis by microhaplotype sequencing of single-source DNA and two-person DNA mixtures. Forensic Sci. Int. Genet. 58, 102689. 10.1016/j.fsigen.2022.102689 35316721

[B6] Baine I. Hui P. (2019). Practical applications of DNA genotyping in diagnostic pathology. Expert Rev. Mol. Diagnostics 19 (2), 175–188. 10.1080/14737159.2019.1568874 30638393

[B7] Ballantyne K. N. Keerl V. Wollstein A. Choi Y. Zuniga S. B. Ralf A. (2012). A new future of forensic Y-chromosome analysis: rapidly mutating Y-STRs for differentiating male relatives and paternal lineages. Forensic Sci. Int. Genet. 6 (2), 208–218. 10.1016/j.fsigen.2011.04.017 21612995

[B8] Ballard D. Winkler-Galicki J. Wesoły J. (2020). Massive parallel sequencing in forensics: advantages, issues, technicalities, and prospects. Int. J. Leg. Med. 134 (4), 1291–1303. 10.1007/s00414-020-02294-0 PMC7295846 32451905

[B9] Becker D. Rodig H. Augustin C. Edelmann J. Götz F. Hering S. (2008). Population genetic evaluation of eight X-chromosomal short tandem repeat loci using Mentype Argus X-8 PCR amplification kit. Forensic Sci. Int. Genet. 2 (1), 69–74. 10.1016/j.fsigen.2007.08.013 19083792

[B10] Bertoglio B. Grignani P. Di Simone P. Polizzi N. De Angelis D. Cattaneo C. (2020). Disaster victim identification by kinship analysis: the Lampedusa October 3rd, 2013 shipwreck. Forensic Sci. Int. Genet. 44, 102156. 10.1016/j.fsigen.2019.102156 31707115

[B11] Børsting C. Mikkelsen M. Morling N. (2012). Kinship analysis with diallelic SNPs–Experiences with the SNP for ID multiplex in an ISO17025 accreditated laboratory. Transfus. Med. Hemotherapy 39 (3), 195–201. 10.1159/000338957 PMC3375138 22851935

[B12] Børsting C. Morling N. (2015). Next generation sequencing and its applications in forensic genetics. Forensic Sci. Int. Genet. 18, 78–89. 10.1016/j.fsigen.2015.02.002 25704953

[B13] Budowle B. Adamowicz M. Aranda X. G. Barna C. Chakraborty R. Cheswick D. (2005a). Twelve short tandem repeat loci Y chromosome haplotypes: genetic analysis on populations residing in North America. Forensic Sci. Int. 150 (1), 1–15. 10.1016/j.forsciint.2005.01.010 15837004

[B14] Budowle B. Bieber F. R. Eisenberg A. J. (2005b). Forensic aspects of mass disasters: strategic considerations for DNA-based human identification. Leg. Med. (Tokyo, Jpn.) 7 (4), 230–243. 10.1016/j.legalmed.2005.01.001 15975517

[B15] Butler J. M. (2007). Short tandem repeat typing technologies used in human identity testing. Biotechniques 43 (4). Sii-Sv. 10.2144/000112582 18019344

[B16] Butler J. M. Buel E. Crivellente F. McCord B. R. (2004). Forensic DNA typing by capillary electrophoresis using the ABI Prism 310 and 3100 genetic analyzers for STR analysis. Electrophoresis 25 (10‐11), 1397–1412. 10.1002/elps.200305822 15188225

[B17] Chen L. Wan Y. Yang T. Zhang Q. Zeng Y. Zheng S. (2023a). Bibliometric and visual analysis of single-cell sequencing from 2010 to 2022. Front. Genet. 14, 1285599. 10.3389/fgene.2023.1285599 38274109 PMC10808606

[B18] Chen N. B. Nedoluzhko A. (2023). Ancient DNA: the past for the future. Bmc Genomics 24 (1), 309. 10.1186/s12864-023-09396-0 37291482 PMC10251542

[B19] Chen Z. Ding C. Gu Y. He Y. Chen B. Zheng S. (2023b). Association between gut microbiota and hepatocellular carcinoma from 2011 to 2022: bibliometric analysis and global trends. Front. Oncol. 13, 1120515. 10.3389/fonc.2023.1120515 37064156 PMC10098157

[B20] Consortium G. P. Abecasis G. R. Altshuler D. Auton A. Brooks L. D. Durbin R. M. (2010). A map of human genome variation from population scale sequencing. Nature 467 (7319), 1061–1073. 10.1038/nature09534 20981092 PMC3042601

[B21] Court D. S. (2018). Forensic genealogy: some serious concerns. Forensic Sci. Int. Genet. 36, 203–204. 10.1016/j.fsigen.2018.07.011 30048922

[B22] Cui W. Chen M. Yang Y. Cai M. Lan Q. Xie T. (2023). Applications of 1993 single nucleotide polymorphism loci in forensic pairwise kinship identifications and inferences. Forensic Sci. Int. Genet. 65, 102889. 10.1016/j.fsigen.2023.102889 37247510

[B23] Dausset J. (1958). Iso-leuko-antibodies. Acta Haematol. 20 (1-4), 156–166. 10.1159/000205478 13582558

[B24] Davenport L. Devesse L. Syndercombe Court D. Ballard D. (2023). Forensic identity SNPs: characterisation of flanking region variation using massively parallel sequencing. Forensic Sci. Int. Genet. 64, 102847. 10.1016/j.fsigen.2023.102847 36863275

[B25] de la Puente M. Ruiz-Ramírez J. Ambroa-Conde A. Xavier C. Amigo J. Casares de Cal M. (2020). Broadening the applicability of a custom multi-platform panel of microhaplotypes: bio-geographical ancestry inference and expanded reference data. Front. Genet. 11, 581041. 10.3389/fgene.2020.581041 33193704 PMC7606911

[B26] Devesse L. Ballard D. Davenport L. Riethorst I. Mason-Buck G. Syndercombe Court D. (2018). Concordance of the ForenSeq™ system and characterisation of sequence-specific autosomal STR alleles across two major population groups. Forensic Sci. Int. Genet. 34, 57–61. 10.1016/j.fsigen.2017.10.012 29413636

[B27] Diepenbroek M. Bayer B. Anslinger K. (2021). Pushing the boundaries: forensic DNA phenotyping challenged by single-cell sequencing. Genes (Basel) 12 (9), 1362. 10.3390/genes12091362 34573344 PMC8466929

[B28] Dykes D. D. Polesky H. F. (1976). The usefulness of serum protein and erythrocyte enzyme polymorphisms in paternity testing. Am. J. Clin. pathology 65 (6), 982–986. 10.1093/ajcp/65.6.982 937253

[B29] Edge M. D. Coop G. (2020). Donnelly (1983) and the limits of genetic genealogy. Theor. Popul. Biol. 133, 23–24. 10.1016/j.tpb.2019.08.002 31430435 PMC7024661

[B30] Ertürk M. S. Fitzpatrick C. Press M. Wein L. M. (2022). Analysis of the genealogy process in forensic genetic genealogy. J. Forensic Sci. 67 (6), 2218–2229. 10.1111/1556-4029.15127 36059116 PMC9826014

[B31] Farash K. Hanson E. K. Ballantyne J. (2018). Single source DNA profile recovery from single cells isolated from skin and fabric from touch DNA mixtures in mock physical assaults. Sci. Justice 58 (3), 191–199. 10.1016/j.scijus.2017.12.006 29685301

[B32] Feng Y. Zhao Y. Lu X. Li H. Zhao K. Shi M. (2024). Forensic analysis and sequence variation of 133 STRs in the Hakka population. Front. Genet. 15, 1347868. 10.3389/fgene.2024.1347868 38317659 PMC10839782

[B33] Fierer N. Lauber C. L. Zhou N. McDonald D. Costello E. K. Knight R. (2010). Forensic identification using skin bacterial communities. Proc. Natl. Acad. Sci. U. S. A. 107 (14), 6477–6481. 10.1073/pnas.1000162107 20231444 PMC2852011

[B34] Frontanilla T. S. Valle-Silva G. Ayala J. Mendes-Junior C. T. (2022). Open-access worldwide population STR database constructed using high-coverage massively parallel sequencing data obtained from the 1000 genomes project. Genes (Basel) 13 (12), 2205. 10.3390/genes13122205 36553472 PMC9778533

[B35] Ge J. Budowle B. (2021). Forensic investigation approaches of searching relatives in DNA databases. J. Forensic Sci. 66 (2), 430–443. 10.1111/1556-4029.14615 33136341

[B36] Gettings K. B. Borsuk L. A. Ballard D. Bodner M. Budowle B. Devesse L. (2017). STRSeq: a catalog of sequence diversity at human identification Short Tandem Repeat loci. Forensic Sci. Int. Genet. 31, 111–117. 10.1016/j.fsigen.2017.08.017 28888135 PMC7304526

[B37] Gill P. Gusmão L. Haned H. Mayr W. Morling N. Parson W. (2012). DNA commission of the International Society of Forensic Genetics: recommendations on the evaluation of STR typing results that may include drop-out and/or drop-in using probabilistic methods. Forensic Sci. Int. Genet. 6 (6), 679–688. 10.1016/j.fsigen.2012.06.002 22864188 PMC4689582

[B38] Glynn C. L. (2022). Bridging disciplines to form a new one: the emergence of forensic genetic genealogy. Genes 13 (8), 1381. 10.3390/genes13081381 36011291 PMC9407302

[B39] Goedbloed M. Vermeulen M. Fang R. X. N. Lembring M. Wollstein A. Ballantyne K. (2009). Comprehensive mutation analysis of 17 Y-chromosomal short tandem repeat polymorphisms included in the AmpF*l*STR® Yfiler® PCR amplification kit. Int. J. Leg. Med. 123 (6), 471–482. 10.1007/s00414-009-0342-y PMC2766043 19322579

[B40] Greytak E. M. Moore C. Armentrout S. L. (2019). Genetic genealogy for cold case and active investigations. Forensic Sci. Int. 299, 103–113. 10.1016/j.forsciint.2019.03.039 30991209

[B41] Hirschfeld J. Jonsson B. Rasmuson M. (1960). Inheritance of a new group-specific system demonstrated in normal human sera by means of an immuno-electrophoretic technique. Nature 185 (4717), 931–932. 10.1038/185931b0 14402002

[B42] Hofreiter M. Sneberger J. Pospisek M. Vanek D. (2021). Progress in forensic bone DNA analysis: lessons learned from ancient DNA. Forensic Sci. Int. Genet. 54, 102538. 10.1016/j.fsigen.2021.102538 34265517

[B43] Huff C. D. Witherspoon D. J. Simonson T. S. Xing J. Watkins W. S. Zhang Y. (2011). Maximum-likelihood estimation of recent shared ancestry (ERSA). Genome Res. 21 (5), 768–774. 10.1101/gr.115972.110 21324875 PMC3083094

[B44] Huffman K. Ballantyne J. (2022). Probabilistic genotyping of single cell replicates from mixtures involving first-degree relatives prevents the false inclusions of non-donor relatives. Genes (Basel) 13 (9), 1658. 10.3390/genes13091658 36140825 PMC9498535

[B45] Huffman K. Ballantyne J. (2023). Single cell genomics applications in forensic science: current state and future directions. iScience 26 (11), 107961. 10.1016/j.isci.2023.107961 37876804 PMC10590970

[B46] Kaiser J. (2018). Forensic genealogy comes of age. Science 362 (6421), 1348.

[B47] Kayser M. (2017). Forensic use of Y-chromosome DNA: a general overview. Hum. Genet. 136 (5), 621–635. 10.1007/s00439-017-1776-9 28315050 PMC5418305

[B48] Khan R. Mittelman D. (2018). Consumer genomics will change your life, whether you get tested or not. Genome Biol. 19 (1), 120–124. 10.1186/s13059-018-1506-1 30124172 PMC6100720

[B49] Kidd K. Pakstis A. Speed W. Lagace R. Chang J. Wootton S. (2013). Microhaplotype loci are a powerful new type of forensic marker. Forensic Sci. Int. Genet. Suppl. Ser. 4 (1), e123–e124. 10.1016/j.fsigss.2013.10.063

[B50] King T. E. Jobling M. A. (2009). What's in a name? Y chromosomes, surnames and the genetic genealogy revolution. Trends Genet. 25 (8), 351–360. 10.1016/j.tig.2009.06.003 19665817

[B51] Klein R. J. Zeiss C. Chew E. Y. Tsai J.-Y. Sackler R. S. Haynes C. (2005). Complement factor H polymorphism in age-related macular degeneration. Science 308 (5720), 385–389. 10.1126/science.1109557 15761122 PMC1512523

[B52] Kling D. Dell’Amico B. Tillmar A. O. (2015). FamLinkX–implementation of a general model for likelihood computations for X-chromosomal marker data. Forensic Sci. Int. Genet. 17, 1–7. 10.1016/j.fsigen.2015.02.007 25771099

[B53] Kling D. Phillips C. Kennett D. Tillmar A. (2021). Investigative genetic genealogy: current methods, knowledge and practice. Forensic Sci. Int. Genet. 52, 102474. 10.1016/j.fsigen.2021.102474 33592389

[B54] Kruglyak L. (2005). Power tools for human genetics. Nat. Genet. 37 (12), 1299–1300. 10.1038/ng1205-1299 16314858

[B55] Kureshi A. Li J. Wen D. Sun S. Yang Z. Zha L. (2020). Construction and forensic application of 20 highly polymorphic microhaplotypes. R. Soc. Open Sci. 7 (5), 191937. 10.1098/rsos.191937 32537197 PMC7277291

[B56] Kurosaki K. Matsushita T. Ueda S. (1993). Individual DNA identification from ancient human remains. Am. J. Hum. Genet. 53 (3), 638–643. 8352274 PMC1682433

[B57] Landsteiner K. (1900). Zur Kenntnis der antifermentativen, lytischen und agglutinierenden Wirkungen des Blutseruns und der Lymphe. Z. Bakteriol. 27, 357–362.

[B58] Larue B. L. Ge J. Y. King J. L. Budowle B. (2012). A validation study of the Qiagen Investigator DIPplex® kit; an INDEL-based assay for human identification. Int. J. Leg. Med. 126 (4), 533–540. 10.1007/s00414-012-0667-9 22249274

[B59] Lei G. Liu F. Liu P. Zhou Y. Jiao T. Dang Y.-H. (2019). Worldwide tendency and focused research in forensic anthropology: a bibliometric analysis of decade (2008–2017). Leg. Med. 37, 67–75. 10.1016/j.legalmed.2019.01.008 30716583

[B60] Liu J. Wang J. Zhang X. Li Z. Yun K. Liu Z. (2017). A mixture detection method based on separate amplification using primer specific alleles of INDELs-a study based on two person's DNA mixture. J. Forensic Leg. Med. 46, 30–36. 10.1016/j.jflm.2017.01.002 28119211

[B61] Liu Q. Ma G. Du Q. Lu C. Fu L. Wang Q. (2020). Development of an NGS panel containing 42 autosomal STR loci and the evaluation focusing on secondary kinship analysis. Int. J. Leg. Med. 134, 2005–2014. 10.1007/s00414-020-02295-z 32314064

[B62] Loreille O. Ratnayake S. Bazinet A. L. Stockwell T. B. Sommer D. D. Rohland N. (2018). Biological sexing of a 4000-year-old Egyptian mummy head to assess the potential of nuclear DNA recovery from the most damaged and limited forensic specimens. Genes (Basel) 9 (3), 135. 10.3390/genes9030135 29494531 PMC5867856

[B63] Majumder M. A. Guerrini C. J. McGuire A. L. (2021). Direct-to-consumer genetic testing: value and risk. Annu. Rev. Med. 72, 151–166. 10.1146/annurev-med-070119-114727 32735764

[B64] Martín P. de Simón L. F. Luque G. Farfán M. J. Alonso A. (2014). Improving DNA data exchange: validation studies on a single 6 dye STR kit with 24 loci. Forensic Sci. Int. Genet. 13, 68–78. 10.1016/j.fsigen.2014.07.002 25082138

[B65] Martínez I. Muller C. E. Walter J. (2013). Long-term temporal analysis of the human fecal microbiota revealed a stable core of dominant bacterial species. PLoS One 8 (7), e69621. 10.1371/journal.pone.0069621 23874976 PMC3712949

[B66] Mateen R. M. Sabar M. F. Hussain S. Parveen R. Hussain M. (2021). Familial DNA analysis and criminal investigation: usage, downsides and privacy concerns. Forensic Sci. Int. 318, 110576. 10.1016/j.forsciint.2020.110576 33234348

[B67] Mayr W. Brinkmann B. Rand S. (1991). Paternity testing—*quo vadis*? Blood Rev. 5 (1), 51–54. 10.1016/0268-960X(91)90008-Z 2032028

[B68] Mill J. Dempster E. Caspi A. Williams B. Moffitt T. Craig I. (2006). Evidence for monozygotic twin (MZ) discordance in methylation level at two CpG sites in the promoter region of the catechol-O-methyltransferase (COMT) gene. Am. J. Med. Genet. B Neuropsychiatr. Genet. 141b (4), 421–425. 10.1002/ajmg.b.30316 16583437

[B69] Murphy E. (2018). Law and policy oversight of familial searches in recreational genealogy databases. Forensic Sci. Int. 292, e5–e9. 10.1016/j.forsciint.2018.08.027 30287164

[B70] Nothnagel M. Schmidtke J. Krawczak M. (2010). Potentials and limits of pairwise kinship analysis using autosomal short tandem repeat loci. Int. J. Leg. Med. 124, 205–215. 10.1007/s00414-009-0413-0 20143081

[B71] Oldoni F. Bader D. Fantinato C. Wootton S. C. Lagacé R. Kidd K. K. (2020). A sequence-based 74plex microhaplotype assay for analysis of forensic DNA mixtures. Forensic Sci. Int. Genet. 49, 102367. 10.1016/j.fsigen.2020.102367 32919300

[B72] Oldoni F. Kidd K. K. Podini D. (2019). Microhaplotypes in forensic genetics. Forensic Sci. Int. Genet. 38, 54–69. 10.1016/j.fsigen.2018.09.009 30347322

[B73] Oldoni F. Podini D. (2019). Forensic molecular biomarkers for mixture analysis. Forensic Sci. Int. Genet. 41, 107–119. 10.1016/j.fsigen.2019.04.003 31071519

[B74] Pajnič I. Z. Geršak Ž. M. Leskovar T. Črešnar M. (2023). Kinship analysis of 5th-to 6th-century skeletons of Romanized indigenous people from the Bled–Pristava archaeological site. Forensic Sci. Int. Genet. 65, 102886. 10.1016/j.fsigen.2023.102886 37137206

[B75] Phillips C. (2018). The Golden State Killer investigation and the nascent field of forensic genealogy. Forensic Sci. Int. Genet. 36, 186–188. 10.1016/j.fsigen.2018.07.010 30041097

[B76] Phillips C. Amigo J. Tillmar A. O. Peck M. A. de la Puente M. Ruiz-Ramírez J. (2020). A compilation of tri-allelic SNPs from 1000 Genomes and use of the most polymorphic loci for a large-scale human identification panel. Forensic Sci. Int. Genet. 46, 102232. 10.1016/j.fsigen.2020.102232 31986343

[B77] Phillips C. Fang R. Ballard D. Fondevila M. Harrison C. Hyland F. (2007). Evaluation of the Genplex SNP typing system and a 49plex forensic marker panel. Forensic Sci. Int. Genet. 1 (2), 180–185. 10.1016/j.fsigen.2007.02.007 19083752

[B78] Phillips C. Gettings K. B. King J. L. Ballard D. Bodner M. Borsuk L. (2018). The devil's in the detail": release of an expanded, enhanced and dynamically revised forensic STR Sequence Guide. Forensic Sci. Int. Genet. 34, 162–169. 10.1016/j.fsigen.2018.02.017 29486434

[B79] Pu Y. Chen P. Zhu J. Jiang Y. Li Q. Feng T. (2017). Microhaplotype: ability of personal identification and being ancestry informative marker. Forensic Sci. Int. Genet. Suppl. Ser. 6, e442–e444. 10.1016/j.fsigss.2017.09.144

[B80] Queller D. C. Strassmann J. E. Hughes C. R. (1993). Microsatellites and kinship. Trends Ecol. Evol. 8 (8), 285–288. 10.1016/0169-5347(93)90256-O 21236170

[B81] Ralf A. Lubach D. Kousouri N. Winkler C. Schulz I. Roewer L. (2020). Identification and characterization of novel rapidly mutating Y-chromosomal short tandem repeat markers. Hum. Mutat. 41 (9), 1680–1696. 10.1002/humu.24068 32579758

[B82] Ralf A. Zandstra D. Weiler N. van Ijcken W. F. J. Sijen T. Kayser M. (2021). RMplex: an efficient method for analyzing 30 Y-STRs with high mutation rates. Forensic Sci. Int. Genet. 55, 102595. 10.1016/j.fsigen.2021.102595 34543845

[B83] Ram N. Guerrini C. J. McGuire A. L. (2018). Genealogy databases and the future of criminal investigation. Science 360 (6393), 1078–1079. 10.1126/science.aau1083 29880677 PMC6542732

[B84] Ram N. Roberts J. L. (2019). Forensic genealogy and the power of defaults. Nat. Biotechnol. 37 (7), 707–708. 10.1038/s41587-019-0172-5 31189937

[B85] Reis-Filho J. S. (2009). Next-generation sequencing. Breast cancer Res. 11 (3), S12–S17. 10.1186/bcr2431 20030863 PMC2797692

[B86] Saiki R. K. Gelfand D. H. Stoffel S. Scharf S. J. Higuchi R. Horn G. T. (1988). Primer-directed enzymatic amplification of DNA with a thermostable DNA polymerase. Science 239 (4839), 487–491. 10.1126/science.2448875 2448875

[B87] Samuel G. Kennett D. (2020). The impact of investigative genetic genealogy: perceptions of UK professional and public stakeholders. Forensic Sci. Int. Genet. 48, 102366. 10.1016/j.fsigen.2020.102366 32781429

[B88] Scheible M. Loreille O. Just R. Irwin J. (2014). Short tandem repeat typing on the 454 platform: strategies and considerations for targeted sequencing of common forensic markers. Forensic Sci. Int. Genet. 12, 107–119. 10.1016/j.fsigen.2014.04.010 24908576

[B89] Schmedes S. E. Sajantila A. Budowle B. (2016). Expansion of microbial forensics. J. Clin. Microbiol. 54 (8), 1964–1974. 10.1128/jcm.00046-16 26912746 PMC4963497

[B90] Schmedes S. E. Woerner A. E. Budowle B. (2017). Forensic human identification using skin microbiomes. Appl. Environ. Microbiol. 83 (22), e01672–17. 10.1128/aem.01672-17 28887423 PMC5666146

[B91] Scudder N. Daniel R. Raymond J. Sears A. (2020). Operationalising forensic genetic genealogy in an Australian context. Forensic Sci. Int. 316, 110543. 10.1016/j.forsciint.2020.110543 33152660

[B92] Seo S. B. King J. L. Warshauer D. H. Davis C. P. Ge J. Y. Budowle B. (2013). Single nucleotide polymorphism typing with massively parallel sequencing for human identification. Int. J. Leg. Med. 127 (6), 1079–1086. 10.1007/s00414-013-0879-7 23736940

[B93] Silver H. (1989). Paternity testing. Crit. Rev. Clin. Laboratory Sci. 27 (5), 391–408. 10.3109/10408368909106594 2572239

[B94] Smithies O. (1955). Zone electrophoresis in starch gels: group variations in the serum proteins of normal human adults. Biochem. J. 61 (4), 629–641. 10.1042/bj0610629 13276348 PMC1215845

[B95] Snedecor J. Fennell T. Stadick S. Homer N. Antunes J. Stephens K. (2022). Fast and accurate kinship estimation using sparse SNPs in relatively large database searches. Forensic Sci. Int. Genet. 61, 102769. 10.1016/j.fsigen.2022.102769 36087514

[B96] Sobrino B. Brión M. Carracedo A. (2005). SNPs in forensic genetics:: a review on SNP typing methodologies. Forensic Sci. Int. 154 (2-3), 181–194. 10.1016/j.forsciint.2004.10.020 16182964

[B97] Song W. Zhou S. Yu W. Fan Y. Liang X. (2023). Genetic analysis of 42 Y-STR loci in Han and Manchu populations from the three northeastern provinces in China. BMC genomics 24 (1), 578. 10.1186/s12864-023-09636-3 37770896 PMC10537175

[B98] Southern E. M. (1975). Detection of specific sequences among DNA fragments separated by gel electrophoresis. J. Mol. Biol. 98 (3), 503–517. 10.1016/S0022-2836(75)80083-0 1195397

[B99] Stasi A. Pellegrino A. Wani A. K. Shukla S. (2023). Forty years of research and development on forensic genetics: a bibliometric analysis. Forensic Sci. Int. Genet. 63, 102826. 10.1016/j.fsigen.2023.102826 36640637

[B100] Syndercombe Court D. (2021). Mitochondrial DNA in forensic use. Emerg. Top. Life Sci. 5 (3), 415–426. 10.1042/etls20210204 34374411 PMC8457767

[B101] Tam J. C. W. Chan Y. M. Tsang S. Y. Yau C. I. Yeung S. Y. Au K. K. (2020). Noninvasive prenatal paternity testing by means of SNP‐based targeted sequencing. Prenat. Diagn. 40 (4), 497–506. 10.1002/pd.5595 31674029 PMC7154534

[B102] Tao R. Yang Q. Xia R. Zhang X. Chen A. Li C. (2022). A sequence-based 163plex microhaplotype assay for forensic DNA analysis. Front. Genet. 13, 988223. 10.3389/fgene.2022.988223 36276985 PMC9579316

[B103] Tillmar A. Fagerholm S. A. Staaf J. Sjölund P. Ansell R. (2021). Getting the conclusive lead with investigative genetic genealogy–A successful case study of a 16 year old double murder in Sweden. Forensic Sci. Int. Genet. 53, 102525. 10.1016/j.fsigen.2021.102525 33991867

[B104] Tirosh I. Venteicher A. S. Hebert C. Escalante L. E. Patel A. P. Yizhak K. (2016). Single-cell RNA-seq supports a developmental hierarchy in human oligodendroglioma. Nature 539 (7628), 309–313. 10.1038/nature20123 27806376 PMC5465819

[B105] Tvedebrink T. (2022). Review of the forensic applicability of biostatistical methods for inferring ancestry from autosomal genetic markers. Genes 13 (1), 141. 10.3390/genes13010141 35052480 PMC8774801

[B106] van Dongen J. Gordon S. D. McRae A. F. Odintsova V. V. Mbarek H. Breeze C. E. (2021). Identical twins carry a persistent epigenetic signature of early genome programming. Nat. Commun. 12 (1), 5618. 10.1038/s41467-021-25583-7 34584077 PMC8479069

[B107] Van Eck N. Waltman L. (2010). Software survey: VOSviewer, a computer program for bibliometric mapping. scientometrics 84 (2), 523–538. 10.1007/s11192-009-0146-3 20585380 PMC2883932

[B108] van Oven M. Kayser M. (2009). Updated comprehensive phylogenetic tree of global human mitochondrial DNA variation. Hum. Mutat. 30 (2), E386–E394. 10.1002/humu.20921 18853457

[B109] Venteicher A. S. Tirosh I. Hebert C. Yizhak K. Neftel C. Filbin M. G. (2017). Decoupling genetics, lineages, and microenvironment in IDH-mutant gliomas by single-cell RNA-seq. Science 355 (6332), eaai8478. 10.1126/science.aai8478 28360267 PMC5519096

[B110] von Dungern E. Hirschfeld L. (1962). Concerning heredity of group specific structures of blood. Transfusion 2 (1), 70–74. 10.1111/j.1537-2995.1962.tb00195.x 13888468

[B111] Walker R. H. Crisan D. (1991). DNA technology: the fourth generation in parentage testing. Transfusion 31 (5), 383–385. 10.1046/j.1537-2995.1991.31591263187.x 1675500

[B112] Wang D. Y. Gopinath S. Lagacé R. E. Norona W. Hennessy L. K. Short M. L. (2015). Developmental validation of the GlobalFiler(®) Express PCR Amplification Kit: a 6-dye multiplex assay for the direct amplification of reference samples. Forensic Sci. Int. Genet. 19, 148–155. 10.1016/j.fsigen.2015.07.013 26226223

[B113] Wang F. Song F. Wang X. Song M. Zhou Y. Liu J. (2022a). Mutation analysis for newly suggested 30 Y-STR loci with high mutation rates in Chinese father-son pairs. Sci. Rep. 12 (1), 15680. 10.1038/s41598-022-20014-z 36127390 PMC9489694

[B114] Wang Z. Qin L. Liu J. Jiang L. Zou X. Chen X. (2022b). Forensic nanopore sequencing of microhaplotype markers using QitanTech's QNome. Forensic Sci. Int. Genet. 57, 102657. 10.1016/j.fsigen.2021.102657 34973558

[B115] Warshauer D. H. Lin D. Hari K. Jain R. Davis C. LaRue B. (2013). STRait Razor: a length-based forensic STR allele-calling tool for use with second generation sequencing data. Forensic Sci. International-Genetics 7 (4), 409–417. 10.1016/j.fsigen.2013.04.005 23768312

[B116] Weber-Lehmann J. Schilling E. Gradl G. Richter D. C. Wiehler J. Rolf B. (2014). Finding the needle in the haystack: differentiating "identical" twins in paternity testing and forensics by ultra-deep next generation sequencing. Forensic Sci. Int. Genet. 9, 42–46. 10.1016/j.fsigen.2013.10.015 24528578

[B117] Wei Y. L. Qin C. J. Dong H. Jia J. Li C. X. (2014). A validation study of a multiplex INDEL assay for forensic use in four Chinese populations. Forensic Sci. Int. Genet. 9, e22–e25. 10.1016/j.fsigen.2013.09.002 24090818

[B118] Weir B. S. Anderson A. D. Hepler A. B. (2006). Genetic relatedness analysis: modern data and new challenges. Nat. Rev. Genet. 7 (10), 771–780. 10.1038/nrg1960 16983373

[B119] Wen D. Xing H. Liu Y. Li J. Qu W. He W. (2022). The application of short and highly polymorphic microhaplotype loci in paternity testing and sibling testing of temperature-dependent degraded samples. Front. Genet. 13, 983811. 10.3389/fgene.2022.983811 36226179 PMC9549137

[B120] White L. K. Hesselberth J. R. (2022). Modification mapping by nanopore sequencing. Front. Genet. 13, 1037134. 10.3389/fgene.2022.1037134 36386798 PMC9650216

[B121] Willson J. Roddur M. S. Liu B. Zaharias P. Warnow T. (2022). DISCO: species tree inference using multicopy gene family tree decomposition. Syst. Biol. 71 (3), 610–629. 10.1093/sysbio/syab070 34450658 PMC9016570

[B122] Wu R. Chen H. Li R. Zang Y. Shen X. Hao B. (2021). Pairwise kinship testing with microhaplotypes: can advancements be made in kinship inference with these markers? Forensic Sci. Int. 325, 110875. 10.1016/j.forsciint.2021.110875 34166816

[B123] Xavier C. de la Puente M. Mosquera-Miguel A. Freire-Aradas A. Kalamara V. Vidaki A. (2020). Development and validation of the VISAGE AmpliSeq basic tool to predict appearance and ancestry from DNA. Forensic Sci. Int. Genet. 48, 102336. 10.1016/j.fsigen.2020.102336 32619960

[B124] Xu Q. Wang Z. Kong Q. Wang X. Huang A. Li C. (2022). Evaluating the effects of whole genome amplification strategies for amplifying trace DNA using capillary electrophoresis and massive parallel sequencing. Forensic Sci. Int. Genet. 56, 102599. 10.1016/j.fsigen.2021.102599 34656831

[B125] Xue J. Qu S. Tan M. Xiao Y. Zhang R. Chen D. (2022). An overview of SNP-SNP microhaplotypes in the 26 populations of the 1000 Genomes Project. Int. J. Leg. Med. 136 (5), 1211–1226. 10.1007/s00414-022-02820-2 35397682

[B126] Xue J. Tan M. Zhang R. Chen D. Liu G. Zheng Y. (2023). Evaluation of microhaplotype panels for complex kinship analysis using massively parallel sequencing. Forensic Sci. Int. Genet. 65, 102887. 10.1016/j.fsigen.2023.102887 37209601

[B127] Yang Y. Xie B. Yan J. (2014). Application of next-generation sequencing technology in forensic science. Genomics Proteomics Bioinforma. 12 (5), 190–197. 10.1016/j.gpb.2014.09.001 PMC4411420 25462152

[B128] Yu W. S. Feng Y. S. Kang K. L. Zhang C. Ji A. Q. Ye J. (2022). Screening of highly discriminative microhaplotype markers for individual identification and mixture deconvolution in East Asian populations. Forensic Sci. Int. Genet. 59, 102720. 10.1016/j.fsigen.2022.102720 35594656

[B129] Yuan L. Chen X. Liu Z. Liu Q. Song A. Bao G. (2020). Identification of the perpetrator among identical twins using next-generation sequencing technology: a case report. Forensic Sci. Int. Genet. 44, 102167. 10.1016/j.fsigen.2019.102167 31605960

[B130] Yuan X. Wang X. Lan Q. Li S. Lin Y. Zhao M. (2024). Using two self-developed InDel panels to explore forensic traits and ancestral components in the Hui group. Genomics 116 (1), 110756. 10.1016/j.ygeno.2023.110756 38061479

[B131] Zeng X. P. King J. L. Stoljarova M. Warshauer D. H. LaRue B. L. Sajantila A. (2015). High sensitivity multiplex short tandem repeat loci analyses with massively parallel sequencing. Forensic Sci. International-Genetics 16, 38–47. 10.1016/j.fsigen.2014.11.022 25528025

[B132] Zhang Q. Wang X. Cheng P. Yang S. Li W. Zhou Z. (2022). Complex kinship analysis with a combination of STRs, SNPs, and indels. Forensic Sci. Int. Genet. 61, 102749. 10.1016/j.fsigen.2022.102749 35939875

[B133] Zong C. Lu S. Chapman A. R. Xie X. S. (2012). Genome-wide detection of single-nucleotide and copy-number variations of a single human cell. Science 338 (6114), 1622–1626. 10.1126/science.1229164 23258894 PMC3600412

[B134] Zou X. He G. Liu J. Jiang L. Wang M. Chen P. (2022). Screening and selection of 21 novel microhaplotype markers for ancestry inference in ten Chinese subpopulations. Forensic Sci. Int. Genet. 58, 102687. 10.1016/j.fsigen.2022.102687 35306296

